# Genotypic and phenotypic characterization of the *Sdccag8^Tn(sb-Tyr)2161B.CA1C2Ove^* mouse model

**DOI:** 10.1371/journal.pone.0192755

**Published:** 2018-02-14

**Authors:** Katie Weihbrecht, Wesley A. Goar, Calvin S. Carter, Val C. Sheffield, Seongjin Seo

**Affiliations:** 1 Department of Pediatrics, University of Iowa, Iowa City, lowa, United States of America; 2 Department of Ophthalmology and Visual Sciences, University of Iowa, Iowa City, lowa, United States of America; 3 Institute for Vision Research, University of Iowa, lowa, United States of America; Rutgers University, UNITED STATES

## Abstract

Nephronophthisis-related ciliopathies (NPHP-RC) are a group of disorders that present with end-stage renal failure in childhood/adolescence, kidney cysts, retinal degeneration, and cerebellar hypoplasia. One disorder that shares clinical features with NPHP-RC is Bardet-Biedl Syndrome (BBS). Serologically defined colon cancer antigen 8 (*SDCCAG8*; also known as NPHP10 and BBS16) is an NPHP gene that is also associated with BBS. To better understand the patho-mechanisms of NPHP and BBS caused by loss of SDCCAG8 function, we characterized an SDCCAG8 mouse model (*Sdccag8*^*Tn(sb-Tyr)2161B*.*CA1C2Ove*^) generated by Sleeping Beauty Transposon (SBT)-mediated insertion mutagenesis. Consistent with the previously reported, independent SDCCAG8 mouse models, our mutant mice display pre-axial polydactyly in their hind limbs. In addition, we report patterning defects in the secondary palate, brain abnormalities, as well as neonatal lethality associated with developmental defects in the lung in our mouse model. The neonatal lethality phenotype is genetic background dependent and rescued by introducing 129S6/SvEvTac background. Genetic modifier(s) responsible for this effect were mapped to a region between SNPs rs3714172 and rs3141832 on chromosome 11. While determining the precise genetic lesion in our mouse model, we found that SBT insertion resulted in a deletion of multiple exons from both *Sdccag8* and its neighboring gene *Akt3*. We ascribe the patterning defects in the limb and the secondary palate as well as lung abnormalities to loss of SDCCAG8, while the developmental defects in the brain are likely due to the loss of AKT3. This mouse model may be useful to study features not observed in other SDCCAG8 models but cautions are needed in interpreting data.

## Introduction

The primary cilium, in association with the centrosomes, acts as a sensory organelle in most mammalian cells. Studies during the past two decades have uncovered that mutations disrupting ciliary and centrosomal proteins cause a set of human genetic diseases termed ciliopathies [1, 2]. Nephronophthisis (NPHP) is a leading cause of end-stage renal failure in children and adolescents and most gene products linked to NPHP localize to primary cilia and/or centrosomes, rendering NPHP a ciliopathy [3]. Consistent with the near ubiquitous existence of the primary cilium in the human body, individuals with NPHP often exhibit additional, extra-renal symptoms such as retinal degeneration, cerebellar hypoplasia, and liver fibrosis [4–12]. NPHP with extra-renal symptoms are classified as NPHP-related ciliopathies (NPHP-RC).

Serologically defined colon cancer antigen 8 (*SDCCAG8)* is an NPHP-RC gene (*NPHP10*), with patients displaying retinal and renal abnormalities, obesity, learning disabilities, and recurrent respiratory infections [4, 13, 14]. Aside from a lack of polydactyly, these patients share many of the cardinal features of Bardet-Biedl Syndrome (BBS), a well studied, pleotropic, model ciliopathy disorder. The identification of *SDCCAG8* mutations in several clinically diagnosed BBS patients resulted in *SDCCAG8* being named the sixteenth BBS gene (*BBS16*) [4, 14]. Studies have determined that localization of SDCCAG8 is to the centrosomes and centriolar satellites [4, 15]. In addition, two independent mouse models of SDCCAG8 have been generated using two distinct gene-trap approaches: here we refer to the one generated by the Hildebrandt lab as *Sdccag8*^*Gt(OST40418)Tigm*^ [16, 17] and the one generated by the Shi lab as *Sdccag8*^*tm1e(EUCOMM)Wtsi*^ [18]. These animal models have been instrumental in dissecting the roles of SDCCAG8 *in vivo* [16–18].

Interestingly, considerable variations have been observed within each mouse model as well as between models with respect to the phenotypic expressivity [16–18]. For example, while cleft palate was not observed in the *Sdccag8*^*Gt(OST40418)Tigm*^ model, it was a highly penetrant phenotype in mice homozygous for the *Sdccag8*^*tm1e(EUCOMM)Wtsi*^ allele (94%; 17 out of 18 mutants). Polydactyly was also found as a major phenotypic component (94%; 17 of 18) in the *Sdccag8*^*tm1e(EUCOMM)Wtsi*^ model. However, it was relatively less penetrant (65%; 13 bilateral, 11 unilateral, of 37) in *Sdccag8*^*Gt(OST40418)Tigm*^ mice. Finally, although the precise prevalence was not reported, the vast majority of homozygotic *Sdccag8*^*tm1e(EUCOMM)Wtsi*^ mutant mice died at P0. In contrast, *Sdccag8*^*Gt(OST40418)Tigm*^ mice were obtained at Mendelian ratios at weaning ages. Of note, these two models were generated in two distinct genetic backgrounds: *Sdccag8*^*Gt(OST40418)Tigm*^ in a C57/129SvEv mixed background and *Sdccag8*^*tm1e(EUCOMM)Wtsi*^ in the C57BL/6 background. It is also notable that variable expressivity is a well-recognized characteristic of ciliopathies and the presence of genetic modifiers that influence the expression of disease phenotypes has been suggested [19–23].

Another mouse model of SDCCAG8 is available through the Jackson Laboratory (FVB/N-Sdccag8^Tn(sb-Tyr)2161B.CA1C2Ove^/J; hereafter this allele is referred to as *Sdccag8*^*SBT*^). This mouse model was generated by a Sleeping Beauty Transposon (SBT)-mediated insertion of a gene-trap cassette between exons 12 and 13 of *Sdccag8*. Here, we report characterization of this mouse model and compare phenotypes observed in our mouse model with those seen in other models. We also mapped the genetic modifier(s) that influences neonatal lethality of *Sdccag8* mutants. Finally, we determined the precise genetic lesion of the *Sdccag8*^*SBT*^ allele, which has been incorrectly annotated.

## Materials and methods

### Mouse

*Sdccag8*^*SBT*^ (FVB/N-Sdccag8^Tn(sb-Tyr)2161B.CA1C2Ove^/J) mice were obtained from the Jackson laboratory (stock number: 017598). Mice were housed on a standard 12-hour light/dark cycle and received *ad libitum* access to food and water. This study was carried out in strict accordance with the recommendations in the Guide for the Care and Use of Laboratory Animals of the National Institutes of Health. The protocol was approved by the Animal Care and Use Committee of the University of Iowa (Animal Protocol 5061426).

### PCR genotyping

Genotypes of mice were determined using primer sequences suggested by the Jackson Laboratory and purchased from Integrated DNA Technology (Coralville, IA) (S1 Table). Wild type forward and reverse primers span the SBT insertion site in the intron region between exons 12 and 13 of *Sdccag8*. Mutant forward and reverse primers are specific to the 3’ region of the gene-trap cassette. PCR amplification of *Akt3* and *Sdccag8* exons was performed using standard protocols with primer sequences listed in S2 Table.

### Bone staining and histology

Post-natal day 0 (P0) mice were euthanized by decapitation with a surgical scissors per guidelines from the Office of Animal Care and Use (OACU) of the NIH. Mice were dissected to isolate the secondary palate and the fore and hind limbs. Skin and fat was manually removed from the secondary palate. Limbs were submerged in 70°C water for 30 seconds to facilitate removal of the epidermal layer. Specimens were kept in all following solutions slowly rocking at room temperature. Specimens were fixed in 95% ethanol for 12–48 hours. Ethanol was replaced with Alcian blue staining solution (0.03% Alcian blue (g/ml), 80% ethanol, 20% acetic acid) for 1–3 days. One to two days were long enough for limbs but the secondary palate usually required 3 days for sufficient Alcian blue staining to occur. Alcian blue staining solution was then replaced with 95% ethanol for 6 hours. Ethanol solution was replaced with 2% KOH solution for 12–24 hours. Specimens were then stained in Alizarin red solution (0.03% Alizarin Red (g/ml), 1% KOH, water) for 12–24 hours. Skeletons were cleared in 1% KOH/20% glycerol solution and imaged by Olympus Stereoscope SZX12.

Lung, brain, and kidney were collected and immersed in a solution of 4% paraformaldehyde in PBS. Tissues were fixed overnight at 4°C then embedded in paraffin at the University of Iowa Central Microscopy Research Facility following standard protocol for embryos. Microtome sections were collected at a thickness of 5 μm. Paraffin was melted at 37°C before sections were stained following the standard hematoxylin and eosin (H&E) staining protocol.

### Immunoblotting

Proteins were extracted from mouse tissues by homogenization in a lysis buffer (50 mM HEPES, 150 mM KCl, 1% (vol/vol) Triton X-100, 2 mM MgCl_2_, 1X protease inhibitor (Roche), 0.5 mM DTT) with a Polytron homogenizer, and protein concentrations were measured using a DC Protein Assay kit (Bio-Rad). Lysates were normalized to total protein quantity and loaded onto a 4–12% NuPAGE gel (Invitrogen) for SDS-PAGE and Western blotting following standard protocols. Rabbit polyclonal SDCCAG8 antibody raised against amino acids 119–138 was a gift from Dr. Friedhelm Hildebrandt (SDCCAG8-NR; [4]), and mouse monoclonal β-actin antibody (clone: AC-15) was obtained from Sigma (#A1978).

### Quantitative reverse transcription-PCR (RT-qPCR)

Total RNAs from the brain, kidney, and lung were extracted using TRIzol Reagent (Invitrogen) following manufacturer instructions. Quantitative PCR was performed as previously described [24, 25]. Briefly, 1 μg of total RNA was used for cDNA synthesis using SuperScript III reverse transcriptase (Invitrogen). Quantitative real-time PCR was performed with iQ SYBR Green Supermix (Bio-Rad) and CFX96 Real-Time PCR Detection System (Bio-Rad). *Rpl19* mRNA levels were used for normalization and the ΔΔCt method [26] was used to calculate changes in gene expression. PCR products were confirmed by melt-curve analysis and sequencing. PCR primer sequences are shown in S3 Table.

### Genetic modifier screen

*Sdccag8*^*+/SBT*^ FVB/NJ were crossed to *Sdccag8*^*+/+*^ 129S6/SvEvTac to generate *Sdccag8*^*+/SBT*^ 129S6/FVB F1 mice. These mixed background heterozygous mice were then intercrossed to generate *Sdccag8*^*SBT/SBT*^ 129S6/FVB F2 mice. Mice were classified as “fatalities” if they expired on post-natal day 0 within 8 hours from the point at which they were first discovered. Breeding pairs were checked every morning at 8 AM and again at 4 PM. Mice were classified as “survivors” if they lived to post-natal day 21 or later. Homozygotic mutant mice that died at some point in between P0 and P21 were excluded from this study. Genotypes of mice were determined as described above. The 129SvEv/Tac strain does not have publicly available SNP sequences. Therefore, to identify SNPs around breakpoints, we used the SNP Query tool available from Jackson Lab and compared the known SNPs in FVB/NJ to two available 129 strains: 129S1/SvImJ and 129X1/SvJ. SNPs were preferentially selected when they showed a difference in both 129 strains compared to FVB/NJ. Primers were selected to flank predicted SNPs for PCR amplification (S4 Table). Primers were first tested on both parental strains to ensure viability and SNP differences. Some primer sets allowed for direct Sanger sequencing submission while others required agarose gel clean up followed by Sanger sequencing.

## Results and discussion

### SBT-mediated gene-trap cassette insertion resulted in a recombination deletion of the tail end of *Sdccag8* and neighboring gene *Akt3*

**Fig 1:**
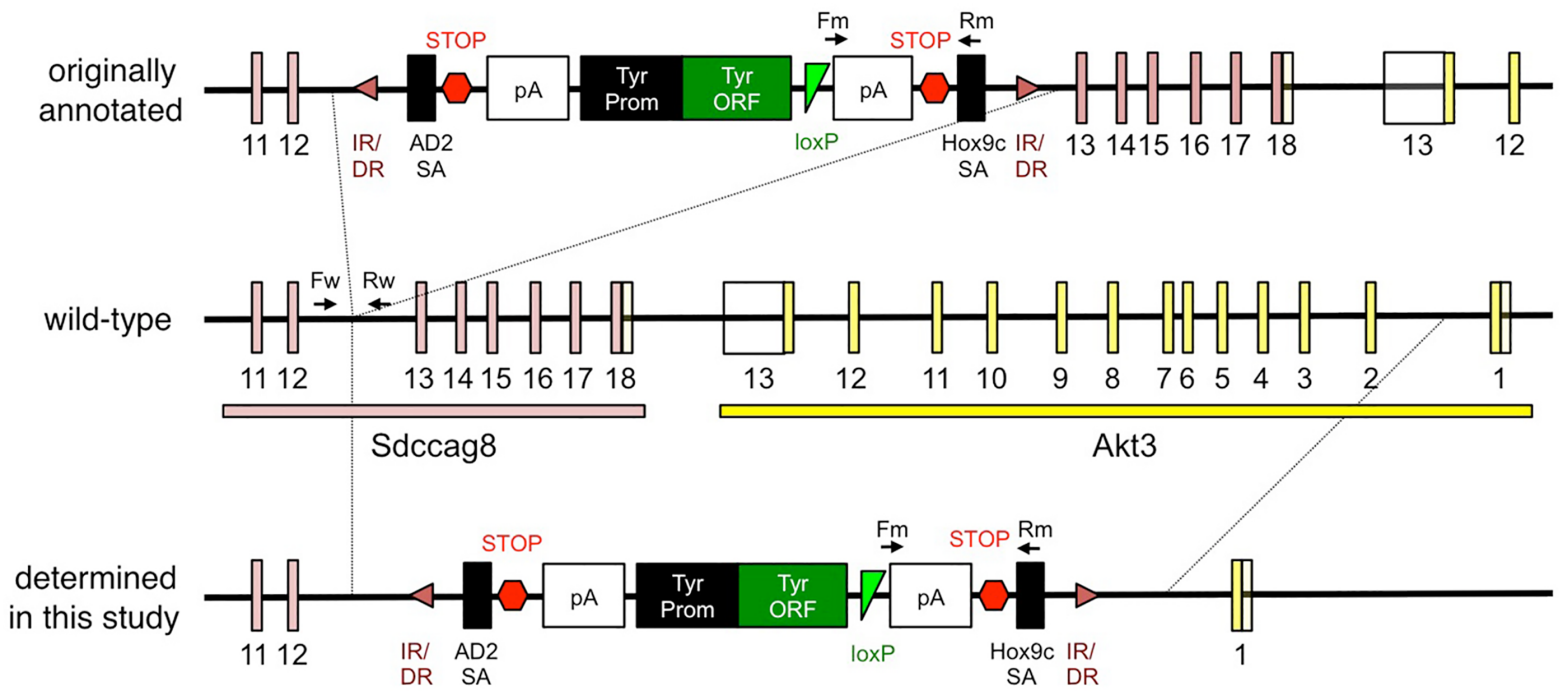
Originally reported and newly determined structure of the *Sdccag8*^*SBT*^ allele. *Top*) originally reported structure of the *Sdccag8*^*SBT*^ allele; *middle*) structure of the wild-type allele; *bottom*) the *Sdccag8*^*SBT*^ allele structure determined in this study. The *Sdccag8* locus is in pink and the *Akt3* locus, in yellow. Contrary to the originally reported structure of the *Sdccag8*^*SBT*^ allele, a deletion that encompasses both *Sdccag8* exons 13–18 and *Akt3* exons 2–13 was identified. IR/DR: Inverted repeat/direct repeat sequence, the SBT recognition site (280 bp); AD2 SA: Adenovirus 2 splice acceptor site; STOP: Stop signal; pA: poly-A sequence; Tyr Prom: Tyrosinase upstream regulatory sequences (2.1 kb from the BALB/c tyrosinase promoter and the first 65 bp of exon 1); Tyr ORF: C57BL/6-derived Tyr^s-J^ cDNA sequence; loxP: Cre recombinase target site; Hox9c SA: Splice acceptor; Fw; WT forward primer site; Rw: WT reverse primer site; Fm: mutant forward primer site; Rm: mutant reverse primer site. All numbers provide an exon number for the corresponding box below it.

**Fig 2:**
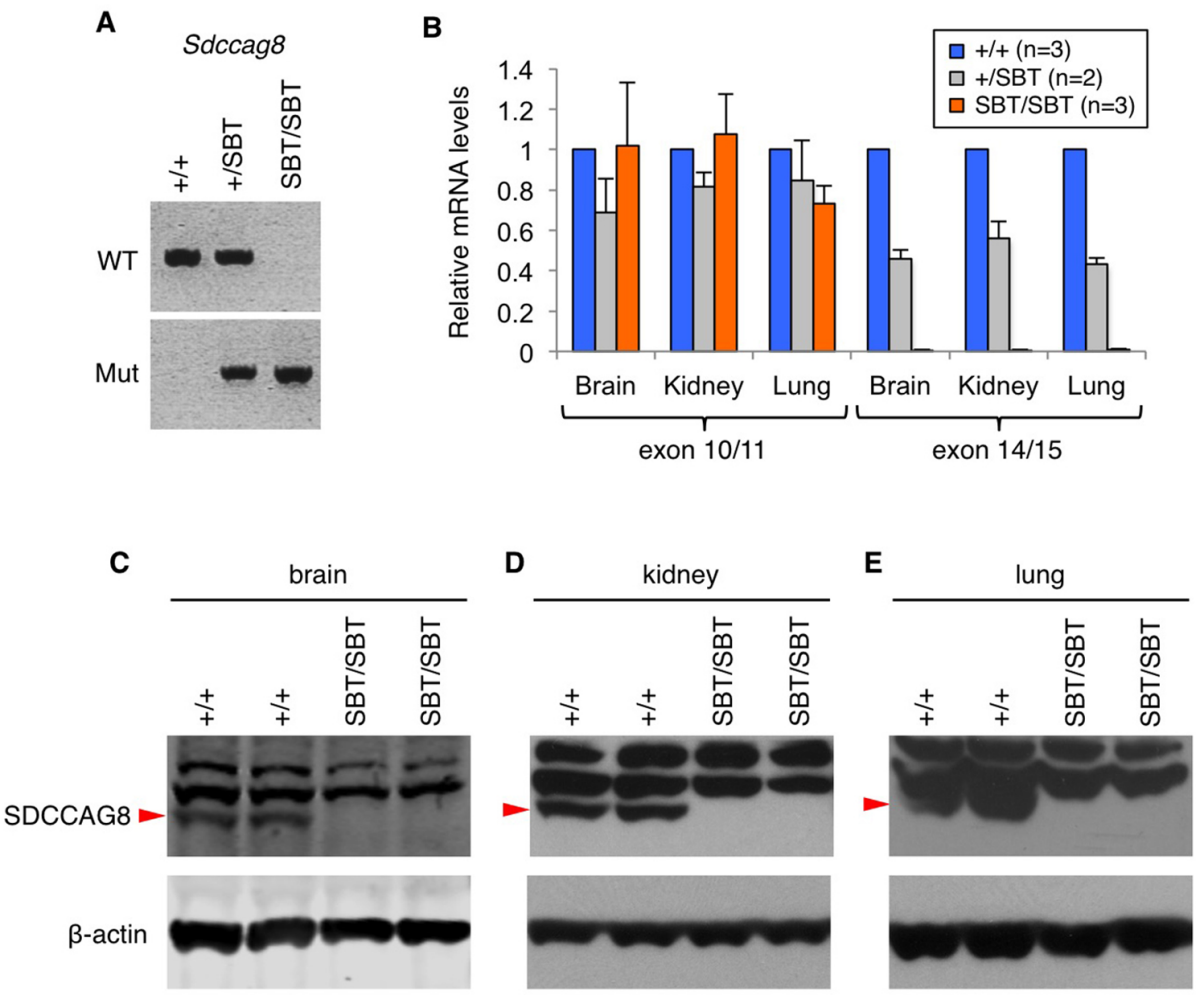
Lack of *Sdccag8* expression in *Sdccag8*^*SBT*^ gene-trap mice. **A)** Representative PCR genotyping results for *Sdccag8*^*+/+*^, *Sdccag8*^*+/SBT*^, *and Sdccag8*^*SBT/SBT*^ mice. Primer pairs Fw+Rw and Fm+Rm detect the presence of wild-type (WT) and mutant (Mut; SBT) alleles, respectively. **B)** qPCR results show the presence of *Sdccag8* mRNAs 5’ of the insertion site but their absence 3’ of the insertion. cDNAs from the brain, kidney, and lung were used for qPCR. Error bars represent standard errors. **C-E)** Immunoblot for SDCCAG8 shows loss of SDCCAG8 in the brain **(C)**, kidney **(D)**, and lung **(E)**. Arrowheads indicate full length SDCCAG8.

The *Sdccag8*^*SBT*^ mutant mouse line was generated by SBT-mediated insertion of the pT2-BART3 transposon transgene [27]. It was reported to the Jackson Laboratory that the gene-trap transgene was inserted between exons 12 and 13 of *Sdccag8* (Fig 1; top), generating a truncated *Sdccag8* mRNA (http://www.jax.org). PCR genotyping with primers spanning the insertion site or primers specific to the transgene show the presence of a normal *Sdccag8* allele in wild-type (WT) and the insertion of the transgene in heterozygotes and homozygotic mutants (Fig 2A; S1 Fig). To determine whether full-length mRNAs encoding SDCCAG8 are produced in mutant mice, primers specific to the upstream (spanning the exon 10–11 boundary) and downstream (spanning the exon 14–15 boundary) (S3 Table) regions of the insertion site were designed, and cDNAs from the brain, kidney, and lung were analyzed by quantitative PCR (qPCR). Our qPCR results indicate that *Sdccag8* transcript levels containing the 5’ region are normal but undetectable at the 3’ end (Fig 2B). The lack of full-length SDCCAG8 was also confirmed by immunoblotting at the protein level (Fig 2C–2E; S2 Fig) and no truncated proteins were detected (S2 Fig). These data indicate that *Sdccag8* mRNAs transcribed from the gene-trap allele do not undergo nonsense-mediated decay but no full-length protein is produced in mutant animals.

**Fig 3:**
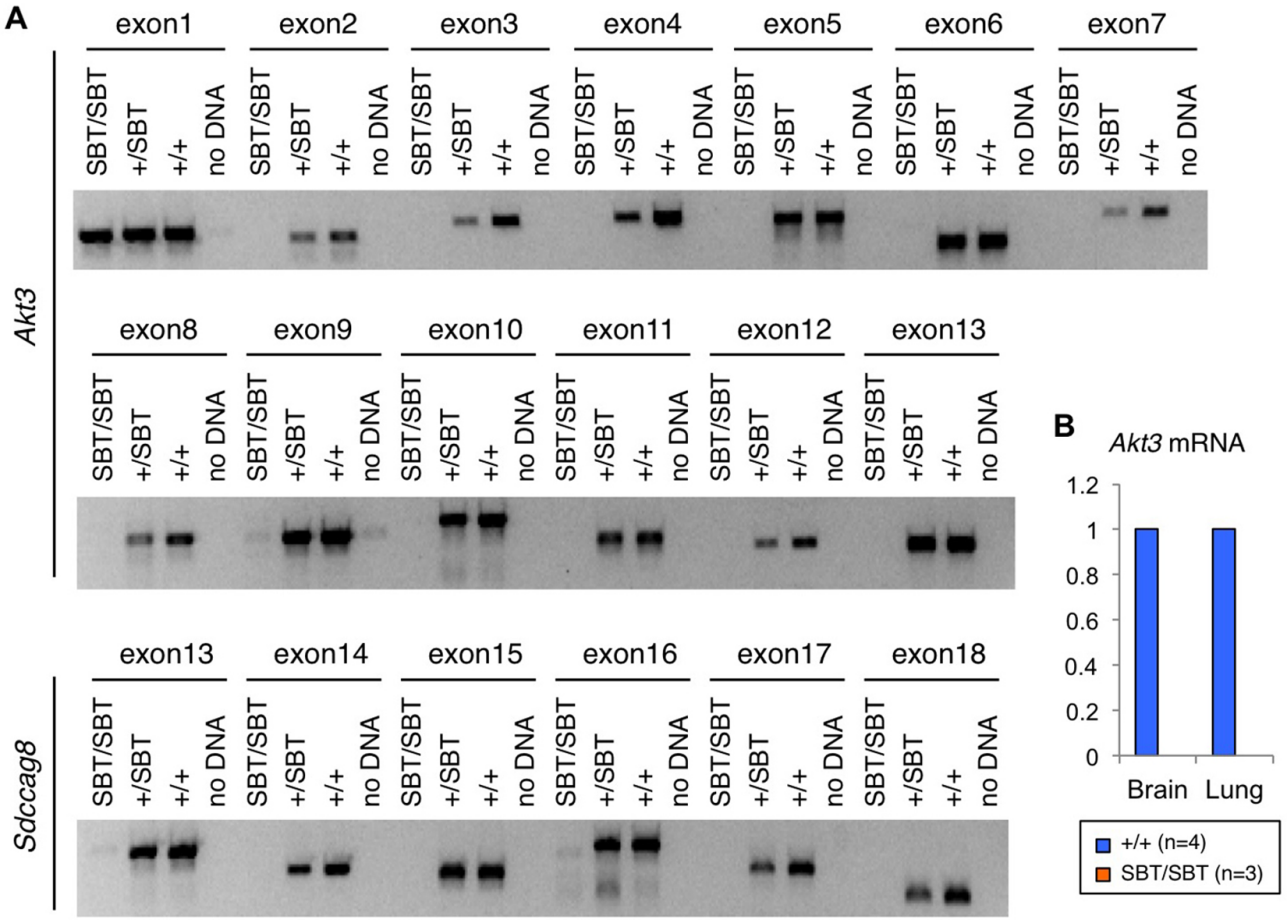
Loss of *Sdccag8* exons 13–18 and *Akt3* exons 2–13 in the *Sdccag8*^*SBT*^ allele. **A)** PCR primers were designed to amplify each exon of *Akt3* and exons 13–18 of *Sdccag8* from mouse genomic DNA. PCR products from *Sdccag8*^*+/+*^, *Sdccag8*^*+/SBT*^, and *Sdccag8*^*SBT/SBT*^ mice were loaded onto an agarose gel. **B**) RT-qPCR with primers spanning exons 3–4 of *Akt3* shows absence of *Akt3* transcripts in *Sdccag8*^*SBT/SBT*^ animals.

The complete absence of transcripts containing exons 14 and 15 is surprising given the levels of transcripts containing exons 5’ of the SBT insertion in mutant animals. To determine whether there were additional genetic lesions in these exons, we designed primers to amplify and sequence exons 13–18 of *Sdccag8* (S2 Table). However, while WT and heterozygous samples showed amplification of these 6 exons, the mutant sample showed no such amplification (Fig 3, S3 Fig), suggesting that *Sdccag8* exons 13–18 are in fact absent in *Sdccag8*^*SBT/SBT*^ mice. To map the boundaries of this genomic deletion, we looked farther downstream of the Sdccag8 locus. We began with amplification and sequencing of the exons of the downstream neighboring gene, *Akt3*. We found that exons 2–13 of *Akt3* (which is tail-to-tail with *Sdccag8*) also failed to amplify in the mutant samples, but showed amplification in WT and heterozygous samples. The lack of *Akt3* transcripts in *Sdccag8*^*SBT/SBT*^ mice was also confirmed by RT-qPCR assays targeting the junction between exons 3–4 of *Akt3* (Fig 3B).

These results indicate that, contrary to the original annotation, the transgene did not simply insert into *Sdccag8* but resulted in a deletion of the region between exon 12 of *Sdccag8* and exon 1 of *Akt3* (Fig 1; bottom). The complete absence of *Sdccag8* mRNAs containing the 3’ region (Fig 2B; exon 14–15), which is unusual for gene-trap alleles, is also consistent with the deletion of those exons on the chromosome.

The most plausible explanation for this large deletion is that instead of a simple insertion occurring, the gene-trap cassette likely inserted in two places, one between exons 12 and 13 of *Sdccag8* and the other between exons 1 and 2 of *Akt3*. Following this double insertion, a recombination event occurred between these two sites that then resulted in a deletion of ~200–250 kb fragment.

### *Sdccag8* loss causes patterning defects in the hind limb and the secondary palate

As mentioned in the Introduction, two independent mouse models of SDCCAG8 have been described (*Sdccag8*^*Gt(OST40418)Tigm*^ [16, 17] and *Sdccag8*^*tm1e(EUCOMM)Wtsi*^ [18]) and considerable variations were observed between these two models. We characterized our mouse model and compared its phenotype with those previously reported, as well as the available *Akt3*^*-/-*^ mouse (Table 1).

**Table 1:** Comparison of phenotypes seen in SDCCAG8 mouse models (*Sdccag8*^*Gt(OST40418)Tigm*^, *Sdccag8*^*tm1e(EUCOMM)Wtsi*^, and *Sdccag8*^*SBT*^) and the *Akt3*^*-/-*^ mouse.

	*Sdccag8*^*Gt(OST40418)Tigm*^	*Sdccag8*^*tm1e(EUCOMM)Wtsi*^	*Akt3*^*-/-*^	*Sdccag8* ^*SBT/SBT*^
Brain	Not reported	22% exhibit an enlarged lateral ventricle. Abnormal neuronal migration.	20–25% reduction in brain-to-body size. Thinning of white matter fiber connections in corpus callosum. Loss of distinction between corpus callosum and surrounding grey matter.	Thinning of white matter/corpus callosum. Decreased anterior commissure size.
Lung	Expression noted in prospective ciliated cells of the developing bronchi and bronchioles. No detrimental phenotype reported.	Not reported	*Akt3* expression noted, no phenotype reported	Paucity of alveoli. Accumulation of blood in the alveolar sacs. Cyanotic appearance indicative of oxygen deprivation.
Palate	Not reported	94% have visible cleft palate	Not reported	No visible cleft. Secondary palate abnormalities seen in the basisphenoid, presphenoid, and the premaxilla.
Polydactyly	Hind limb, pre-axial (65% penetrant, 35% bilateral, 30% unilateral)	94% have pre-axial polydactyly. (laterality not specified, rear or fore not specified)	Not reported	Hind limb, pre-axial (100% penetrant, 100% bilateral)
Background	Mixed (129S6/SvEvBrd, C57BL/6J)	C57BL/6N-A	Mixed (~25% 129/Ola ES, 75% C57BL/6)	FVB/N

**Fig 4:**
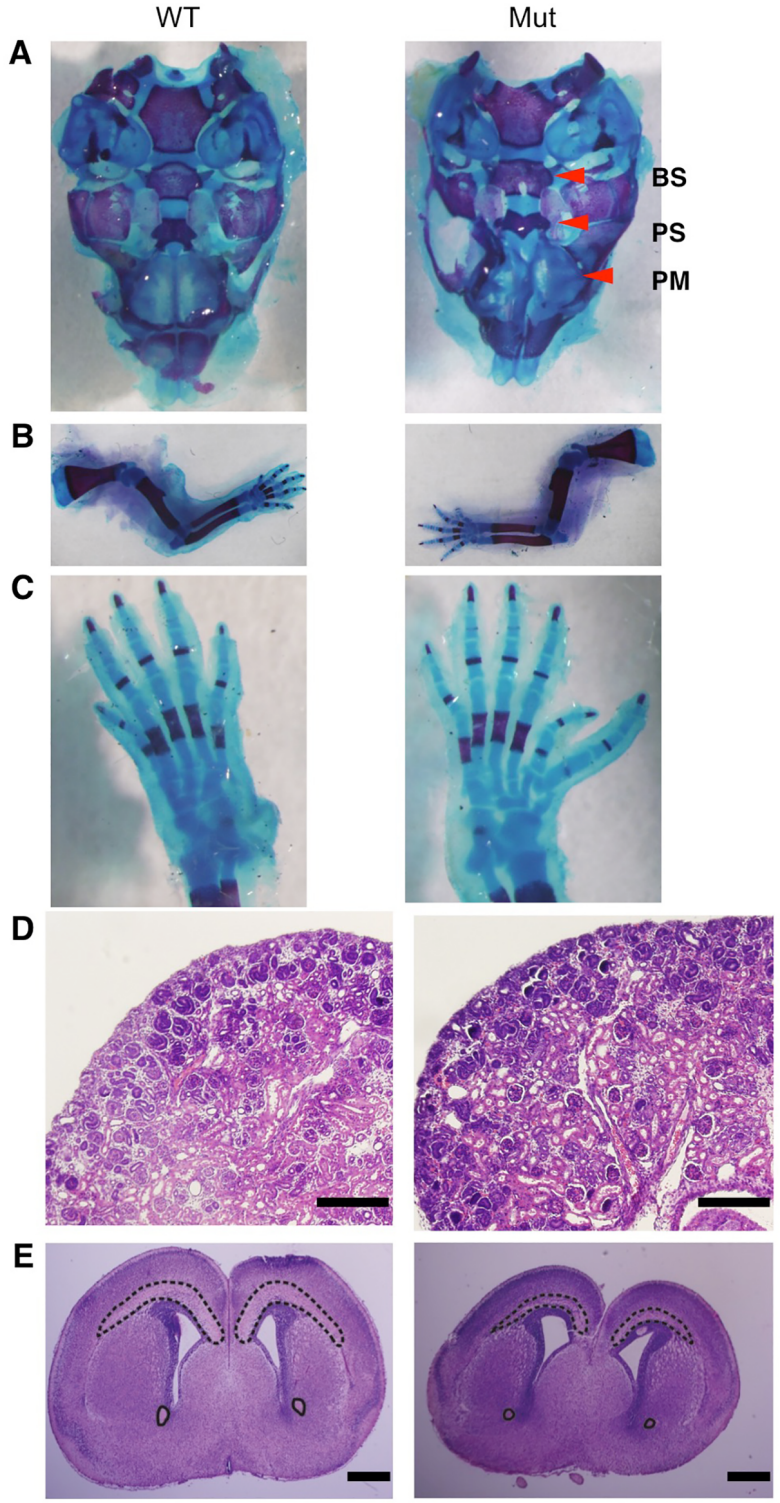
Neonatal *Sdccag8*^*SBT/SBT*^ mice have secondary palate anomalies, pre-axial polydactyly, and brain abnormalities but not cystic kidney. **A)** Alcian blue and Alizarin red staining of the secondary palate. Arrowheads show alterations to the basisphenoid (BS), presphenoid (PS), and premaxilla (PM) regions of the palate. **B)** Alcian blue and Alizarin red staining of the forelimb. **C)** Alcian blue and Alizarin red staining of the hind limb. **D)** H&E staining of kidney sections. Scale bar = 200 μm **E)** H&E-stained brain sections. Anterior commissures are circled and the dashed line highlights the white matter and corpus callosum. Scale bar = 500 μm. In all panels, wild-type (at P0) is shown on the left and *Sdccag8*^*SBT/SBT*^ mutant littermates are on the right.

As for the cleft palate, we were not able to find obvious external clefting in *Sdccag8*^*SBT/SBT*^ mice (n = 47). To investigate whether there are mild patterning defects that are not externally visible, we performed Alcian blue and Alizarin red staining on the secondary palate of P0 mice. Isolation of the secondary palate allowed us to look at the bone and cartilage structure of the premaxilla region, usually associated with visible clefting of the palate. Staining of the secondary palate showed abnormal patterning in mutant mice most noticeable in the basisphenoid, presphenoid, and the premaxilla (Fig 4A; n = 4). However, this abnormal patterning was restricted to minor changes in the pattern of the two sides of the palate coming together. It did not include a failure of the two sides to meeting, and thus did not result in a cleft.

Pre-axial polydactyly was reported in both *Sdccag8*^*Gt(OST40418)Tigm*^ and *Sdccag8*^*tm1e(EUCOMM)Wtsi*^ models with variable penetrance [16–18]. Consistent with the prior studies, polydactyly was observed only in the hind limbs but not in the forelimbs (Fig 4B and 4C). The penetrance of pre-axial polydactyly in our mouse model was 100% (n = 47) and in all cases polydactyly was bilateral. Therefore, our model was closer to the *Sdccag8*^*tm1e(EUCOMM)Wtsi*^ mouse (94% display polydactyly, limbs affected and laterality not reported) with respect to the penetrance [18].

The increased penetrance seen in *Sdccag8*^*SBT/SBT*^ mice may be due to the additional *Akt3* gene deletion. However, to our knowledge, *Akt3* does not have a role in limb bud development and *Akt3* knockout mice do not exhibit polydactyly [28, 29]. In addition, the penetrance of polydactyly in the *Sdccag8*^*tm1e(EUCOMM)Wtsi*^ mouse model, in which the *Akt3* locus is intact, is nearly complete. This suggests that *Akt3* is not likely a contributing factor to the increased penetrance seen in *Sdccag8*^*SBT/SBT*^ mice. A more plausible explanation is that the deletion of 6 coding exons in the *Sdccag8*^*SBT*^ allele makes it a null allele, while *Sdccag8*^*tm1e(EUCOMM)Wtsi*^ and *Sdccag8*^*Gt(OST40418)Tigm*^ are strong hypomorphic alleles that allow a low level expression of normal *Sdccag8* gene.

### Loss of *Sdccag8* does not result in the early development of kidney cysts

It was reported to the Jackson Laboratory (http://www.jax.org) that *Sdccag8*^*SBT/SBT*^ mice have cystic kidneys at P0. While kidney phenotypes were not reported in the *Sdccag8*^*tm1e(EUCOMM)Wtsi*^ model, which also expires at P0, Airik *et al*. reported cyst development in the kidney, first noticeable at P100, in *Sdccag8*^*Gt(OST40418)Tigm*^ mice [16]. In our *Sdccag8*^*SBT*^ colony, we were not able to find cystic kidneys at P0 (Fig 4D). Mutant kidneys were comparable to their wild type and heterozygous littermates. Due to the neonatal lethality, we were not able to examine kidney abnormalities in older animals.

### *Sdccag8*^*SBT/SBT*^ mice display brain abnormalities consistent with *Akt3* loss

Brain abnormalities were reported in both SDCCAG8 deficient (*Sdccag8*^*tm1e(EUCOMM)Wtsi*^) and *Akt3* knockout mice [18, 28, 29]. The *Sdccag8*^*tm1e(EUCOMM)Wtsi*^ mouse demonstrates abnormal neuronal migration in the developing cortex. However, no obvious microcephaly is seen in the brains of mutant animals, suggesting that cortical neurogenesis is near normal in these animals [18]. While little is known about the role of *Akt3*, it has been shown that *Akt3* knockout mice show a 20–25% reduction in the brain size in relation to whole body size due to a decrease in cell size and number [28, 29]. They also show a thinning of the white matter fiber connections in the corpus callosum, as well as a loss of distinction between the corpus callosum and the surrounding grey matter [28].

We found that the corpus callosum and anterior commissures were thinner and there was a deficiency of white matter in *Sdccag8*^*SBT/SBT*^ mice (Fig 4E). These neurological phenotypes are very similar to those seen in *Akt3*^*-/-*^ mice. Therefore, we conclude that the brain abnormalities observed in *Sdccag8*^*SBT/SBT*^ mice are at least partly due to the loss of AKT3 function. The contribution of SDCCAG8 loss to these phenotypes is currently unclear.

### Loss of SDCCAG8 function results in neonatal death in the FVB background, associated with abnormal lung development

One notable difference between the *Sdccag8*^*Gt(OST40418)Tigm*^ and the *Sdccag8*^*tm1e(EUCOMM)Wtsi*^ model is the neonatal lethality phenotype of mutant pups. *Sdccag8*^*Gt(OST40418)Tigm*^ mice are born with a Mendelian ratio and survive to the weaning age [16]. In contrast, although the precise time of death was not reported, homozygous *Sdccag8*^*tm1e(EUCOMM)Wtsi*^ mutant mice die shortly after birth [18]. *Sdccag8*^*SBT/SBT*^ mutant mice are also born at Mendelian ratios (~25%). However, all of them (n> 47) die within 12 hours following birth, indicating that loss of *Sdccag8* is neonatal lethal in the *Sdccag8*^*SBT/SBT*^ mouse model.

**Fig 5:**
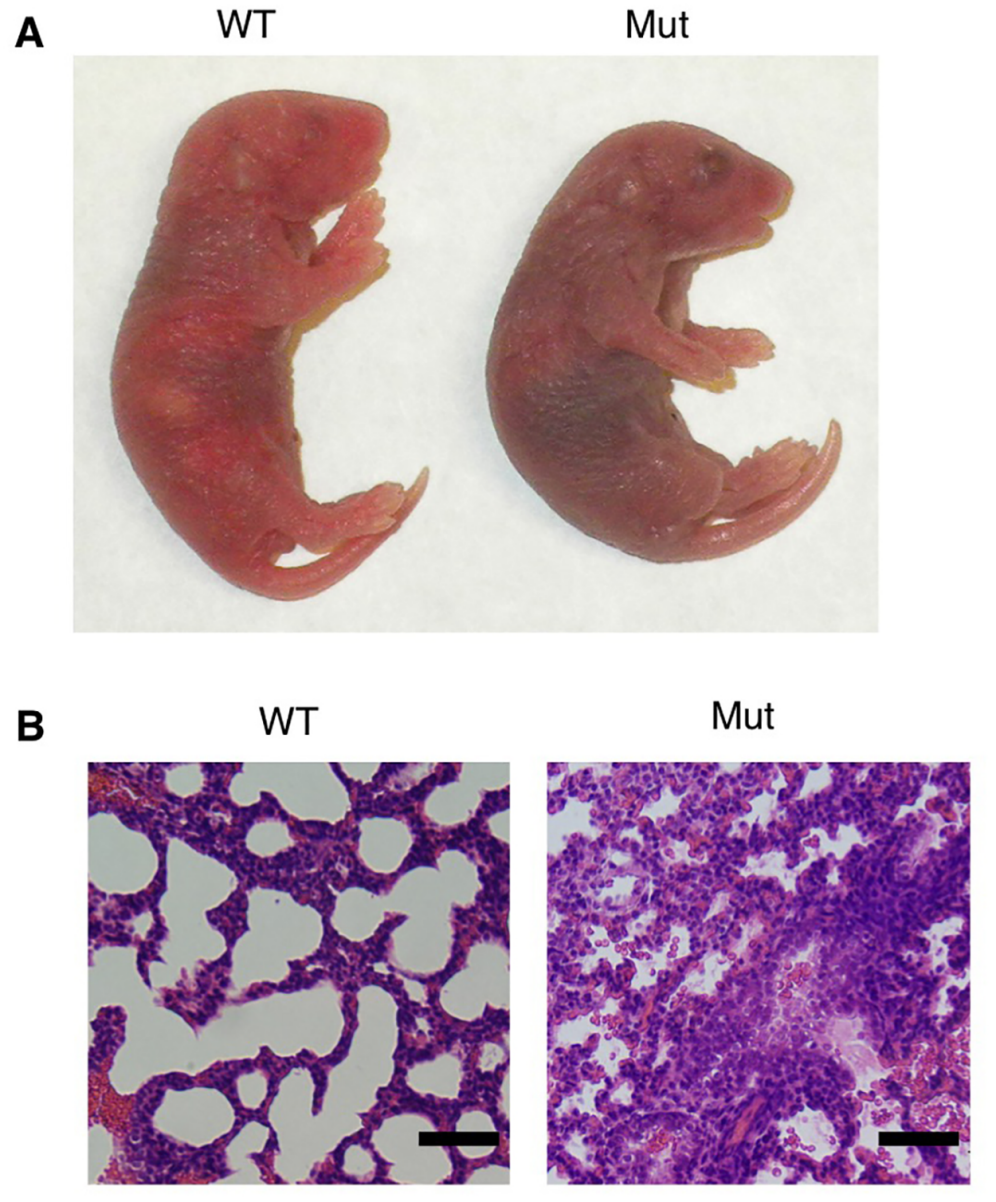
Developmental defects in the *Sdccag8*^*SBT/SBT*^ mutant lung. **A**) *Sdccag8*^*SBT/SBT*^ mice (right) are cyanotic at P0 (before death). A wild-type (WT) littermate is shown on the left. **B**) H&E staining of lung sections from a WT (left) and a mutant littermate (right). Scale bar = 20 μm.

Most *Sdccag8*^*SBT/SBT*^ pups die within 8 hours after birth and all within 12 hours. Failure of suckling is often considered as a cause of neonatal lethality when cleft palate is present. However, despite the presence of some defects in the secondary palate, *Sdccag8*^*SBT/SBT*^ mice do not display obvious clefting externally. Instead, we found that *Sdccag8*^*SBT/SBT*^ mice are cyanotic and gasping, indicative of oxygen deprivation (Fig 5A). Airik *et al*. reported the expression of *Sdccag8* in the prospective ciliated cells of the developing bronchi and bronchioles [16]. Prompted by these findings, we examined the histology of the lung at P0. H&E staining of the lung sections revealed that, compared to littermate controls, the interstitium of *Sdccag8*^*SBT*^ mutant lungs remain thick. Reduction of alveolar airspaces and accumulation of blood in the alveolar sacs was also observed in *Sdccag8*^*SBT/SBT*^ mice (Fig 5B). These findings suggest that the progression from the canalicular stage to the terminal saccular stage during lung development [30] is halted in *Sdccag8*^*SBT/SBT*^ mice. Therefore, although failure of suckling could contribute, our data indicate that developmental defects in the lung and consequent failure of normal gas exchange and oxygen deprivation are likely the main cause of neonatal lethality in *Sdccag8*^*SBT/SBT*^ mice.

Interestingly, human *SDCCAG8* patients diagnosed with BBS have been reported to have recurrent pulmonary infections and poor respiratory function [4, 13, 14]. Although *Akt3* is highly expressed in the lung, absence of *Akt3* is not lethal and no lung phenotypes have been observed in *Akt3*^*-/-*^ animals [29]. This suggests that *Akt3* does not have an essential role in lung development. Based on the known expression pattern of *Sdccag8* in the lung [16], neonatal lethality observed in another SDCCAG8 mouse model (*Sdccag8*^*tm1e(EUCOMM)Wtsi*^) [18], and the lack of a pulmonary phenotype in *Akt3*^*-/-*^ mice [29], we conclude that the developmental defects in the *Sdccag8*^*SBT/SBT*^ mutant lung are due to a loss of *Sdccag8*.

### Genetic contribution from 129S6/SvEvTac provides a protective effect against neonatal lethality

We have generated multiple BBS mouse models, including BBS1, BBS2, BBS4, and BBS6 [31–34]. We have noticed genetic background-dependent neonatal lethality in all of these mouse models; most BBS mutant mice in a 129 background survive normally to the weaning age and beyond, but those that reach weaning age are extremely rare in FVB or C57 backgrounds [35]. The *Sdccag8*^*tm1e(EUCOMM)Wtsi*^ model, which shows neonatal lethality, is on the C57 background [18], and the *Sdccag8*^*Gt(OST40418)Tigm*^ model, which does not show early lethality, is on a C57/129 mixed background [16]. Our *Sdccag8*^*SBT*^ mice are on the FVB background and show neonatal lethality. Based on the correlation between the genetic background and the early lethality phenotype in these mouse models, we hypothesized that there is a genetic modifier(s) that influences the survival of *Sdccag8* mutant pups.

To test this hypothesis and map the modifier(s), we mated *Sdccag8*^*+/SBT*^ FVB/NJ mice with wild-type 129S6/SvEvTac mice and began an intercross of *Sdccag8*^*+/SBT*^ animals (F1) in a FVB/129S6 mixed background. The resulting F2 generation mice were used for phenotyping as well as genetic modifier mapping. While polydactyly was retained in the F2 *Sdccag8*^*SBT/SBT*^ mice at 100% penetrance, we found that adding the 129S6/SvEvTac background resulted in 78% (42 of 54) survival to P21 or later, with only 22% (12 of 54) of F2 pups expiring at P0 similar to their pure strain FVB/NJ counterparts, suggesting the presence of a 129 allele that allows for survival.

To map the modifier, DNA samples from a pilot set of F2 mice, 10 of which died at P0 (fatalities) and 10 of which survived to P21 or later (survivors), were sent to the Jackson lab and genotyped on a custom 150 SNP panel specific to the FVB/NJ and 129S6/SvEvTac lines. Using a Chi Square test of independence, we identified two adjacent SNPs on chromosome 11 which showed a significant genetic association (S5 Table). These SNPs were rs3023266 (p = 0.018) and rs3714172 (p = 0.03). Genotypes at these SNPs were confirmed using Sanger sequencing.

Further analysis was performed on additional SNPs throughout the region surrounding rs3023266 and rs3714172, using the original cohort and an additional 32 mice that survived. Statistical analysis of these SNPs was performed using Chi Square analysis with expected ratios of 1:2:1 FVB:Het:129 versus observed genotypes in the surviving mice. Since introduction of the 129S6 allele promoted survival, we strove to identify an interval lacking FVB/NJ homozygosity in mice that survived. This analysis identified a region of 8 Mb from rs3714172 to rs3141832 that showed a significant genetic skew towards decreased FVB homozygosity (Table 2). There are 162 genes in this 8 Mb region (S6 Table).

**Table 2:** A region between rs3714172 and rs3141832 shows a significant association with survival. Comparison of allele distribution in mice that survive to P21 or later, analyzed using a Chi Square test with expected distribution.

		Survivors			
SNP	Genomic location	FVB	129	Het	p-value
rs26943877	11:56664722	11	11	20	0.954
rs3023266	11:61070650	10	13	19	0.667
rs26956597	11:64233582	9	13	20	0.651
rs26915029	11:72345502	5	11	26	0.129
rs3714172	11:75605462	5	17	20	0.031
rs28214055	11:79737141	5	17	20	0.031
rs29432702	11:82952213	6	17	19	0.046
rs6169904	11:83443872	6	17	19	0.046
rs3141832	11:83580858	6	17	19	0.046
rs27100337	11:89766718	8	18	16	0.028
rs13481176	11:97551944	8	15	19	0.257

We defined our region based upon the hypothesis that 129 provides a protective effect against expiration in survivors and thus we focus on minimizing the number of FVB homozygotes while deviating from expected distribution, rather than simply deviating from expected 1:2:1 distribution. While the rs27100337 SNP has the lowest p-value in this interval, it also has a greatly increased (33% increased from 6 to 8) number of FVB homozygotes.

This region contains an interesting locus, a missense mutation in *Matrix Metalloproteinase-28* (*Mmp28*) at rs6169904. The B6 reference strain and the FVB strain have a shared allele, whereas the 129S6 allele has a p.V342A (NP_536701.1) variant. Although this variation may not be a major change, it is within the 7th exon of *Mmp28*, which is subject to alternative splicing. Furthermore, this variant falls within a haemopexin domain found in isoforms 1 and 3 but not isoform 2 of *Mmp28*. The haemopexin domain can play a role in substrate recognition and it is thought that the alternative splicing of *Mmp28* in this region may alter substrate binding capacity or preference [36]. However, the catalytic activity and substrate specificity of either alternative spice variant of *Mmp28* towards a biologically relevant substrate has not been studied. Under normal conditions, *Mmp28* is expressed at high levels in the lung and promotes epithelial cell survival [37]. It also regulates macrophage polarization and limits macrophage recruitment to tissues [38–40].

In addition to *Mmp28*, all 10 murine genes of the *Schlafen* family are found within the identified region of interest. One SNP studied, rs29432792, is found in the intronic region of *Slfn5*. While little is known about the expression patterns and functions of the individual members, the *Schlafen* (SLFN) family has been implicated in the control of cell proliferation, induction of immune responses, and the regulation of viral replication [41], along with an implication in DDK syndrome, a syndrome which causes implantation defects and early embroynic lethality when females of the DDK inbred mouse strain are mated to many non-DDK strain males [42]. More recently, it has been shown that Schlafen (SLFN) proteins are regulated by interferons and evidence suggests that SLFN proteins play an important role in the anti-neoplastic and growth inhibitory effects of interferons. SLFN1 and SLFN2 have shown upregulation in cystic fibrosis lungs [43] and SLFN5 has an anti-neoplastic effect in renal cell carcinoma [44]. While large changes to any of these proteins is not expected, the genetics of the *Schlafen* family on the FVB/NJ background may make them susceptible to a more extreme phenotype given the right conditions. The loss of SDCCAG8 may affect this family’s ability to complete their normal duties, resulting in death on the FVB/NJ strain, with this extreme phenotype mitigated by having a 129S6/SvEvTac genome across this region.

While having a single copy of 129S6/SvEvTac in this interval greatly increased the odds of survival in *Sdccag8* mice, it should be noted that some mice homozygous for FVB/NJ in this region did survive to P21 or later (Table 2). Therefore, although this region is associated with survival in the presence of the 129S6/SvEvTac background, there may be additional regions that also provide protection from early lethality.

## Conclusions

We show that the *Sdccag8*^*Tn(sb-Tyr)2161B*.*CA1C2Ove*^ mouse displays early neonatal lethality due to abnormal lung development and patterning defects in the secondary palate and the hind limb. We also determined the precise genetic lesion present in this mouse model. Contrary to the original annotation that the pT2-BART3 gene-trap cassette is inserted between the exons 12 and 13 of *Sdccag8*, our study identified a large deletion that encompasses exons 13–18 of *Sdccag8* and exons 2–13 of *Akt3*. We conclude that developmental defects of the secondary palate, hind limbs, and the lung are due to a loss of *Sdccag8*, whereas the brain phenotype is likely due to loss of *Akt3*. We suggest this mouse model may be useful to study the roles of SDCCAG8 *in vivo* but cautions are needed due to the confounding *Akt3* deletion in this model. Finally, we identified a region of interest on chromosome 11 that provides a protective effect against neonatal lethality in *Sdccag8*^*SBT/SBT*^ mice when genomic contribution from the 129S6/SvEvTac background is present.

## Supporting information

S1 Fig Full size agarose gel image depicted in Fig 2A. Red boxes denote DNA samples shown in Fig 2A.
(TIF)

S2 Fig Full size immunoblot images depicted in Fig 2C–2E. (TIF)

S3 Fig Full size agarose gel depicted in Fig 3. (TIF)

S1 Table Genotyping PCR primers for *Sdccag8*^*SBT*^. (DOCX)

S2 Table Primers designed to amplify the exons of interest in *Sdccag8* and *Akt3*. (DOCX)

S3 Table Quantitative real time PCR primers used in this study. (DOCX)

S4 Table Primers designed to amplify SNPs of interest. (DOCX)

S5 Table A custom 150-SNP panel from Jackson Lab showed an association to survival at rs3023266 and rs3714172 (bold/italics). Comparison of allele distribution in mice that died at P0 to mice that survived to P21 or greater as analyzed by a Chi Square test of independence.
(DOCX)

S6 Table List of genes with chromosome position in the 8 Mb region between SNPs rs3714172 and rs3141832. (DOCX)

## References

[1] Gerdes JM, Davis EE, Katsanis N. The vertebrate primary cilium in development, homeostasis, and disease. Cell. 2009;137(1):32–45. Epub 2009/04/07. doi: 10.1016/j.cell.2009.03.023 . 19345185 10.1016/j.cell.2009.03.023 PMC3016012

[2] Badano JL, Mitsuma N, Beales PL, Katsanis N. The ciliopathies: an emerging class of human genetic disorders. Annu Rev Genomics Hum Genet. 2006;7:125–48. doi: 10.1146/annurev.genom.7.080505.115610 . 16722803 10.1146/annurev.genom.7.080505.115610

[3] Hildebrandt F, Otto E. Cilia and centrosomes: a unifying pathogenic concept for cystic kidney disease? Nat Rev Genet. 2005;6(12):928–40. doi: 10.1038/nrg1727 . 16341073 10.1038/nrg1727

[4] Otto EA, Hurd TW, Airik R, Chaki M, Zhou W, Stoetzel C, et al Candidate exome capture identifies mutation of SDCCAG8 as the cause of a retinal-renal ciliopathy. Nature genetics. 2010;42(10):840–50. Epub 2010/09/14. doi: 10.1038/ng.662 . 20835237 10.1038/ng.662 PMC2947620

[5] Olbrich H, Fliegauf M, Hoefele J, Kispert A, Otto E, Volz A, et al Mutations in a novel gene, NPHP3, cause adolescent nephronophthisis, tapeto-retinal degeneration and hepatic fibrosis. Nature genetics. 2003;34(4):455–9. Epub 2003/07/23. doi: 10.1038/ng1216 . 12872122 10.1038/ng1216

[6] Otto E, Hoefele J, Ruf R, Mueller AM, Hiller KS, Wolf MT, et al A gene mutated in nephronophthisis and retinitis pigmentosa encodes a novel protein, nephroretinin, conserved in evolution. American journal of human genetics. 2002;71(5):1161–7. Epub 2002/09/03. doi: 10.1086/344395 . 12205563 10.1086/344395 PMC385091

[7] Otto EA, Loeys B, Khanna H, Hellemans J, Sudbrak R, Fan S, et al Nephrocystin-5, a ciliary IQ domain protein, is mutated in Senior-Loken syndrome and interacts with RPGR and calmodulin. Nature genetics. 2005;37(3):282–8. Epub 2005/02/22. doi: 10.1038/ng1520 . 15723066 10.1038/ng1520

[8] Sayer JA, Otto EA, O'Toole JF, Nurnberg G, Kennedy MA, Becker C, et al The centrosomal protein nephrocystin-6 is mutated in Joubert syndrome and activates transcription factor ATF4. Nature genetics. 2006;38(6):674–81. Epub 2006/05/10. doi: 10.1038/ng1786 . 16682973 10.1038/ng1786

[9] Valente EM, Silhavy JL, Brancati F, Barrano G, Krishnaswami SR, Castori M, et al Mutations in CEP290, which encodes a centrosomal protein, cause pleiotropic forms of Joubert syndrome. Nature genetics. 2006;38(6):623–5. Epub 2006/05/10. doi: 10.1038/ng1805 . 16682970 10.1038/ng1805

[10] Delous M, Baala L, Salomon R, Laclef C, Vierkotten J, Tory K, et al The ciliary gene RPGRIP1L is mutated in cerebello-oculo-renal syndrome (Joubert syndrome type B) and Meckel syndrome. Nature genetics. 2007;39(7):875–81. Epub 2007/06/15. doi: 10.1038/ng2039 . 17558409 10.1038/ng2039

[11] Halbritter J, Porath JD, Diaz KA, Braun DA, Kohl S, Chaki M, et al Identification of 99 novel mutations in a worldwide cohort of 1,056 patients with a nephronophthisis-related ciliopathy. Human genetics. 2013;132(8):865–84. doi: 10.1007/s00439-013-1297-0 . 23559409 10.1007/s00439-013-1297-0 PMC4643834

[12] Wolf MT. Nephronophthisis and related syndromes. Current opinion in pediatrics. 2015;27(2):201–11. doi: 10.1097/MOP.0000000000000194 . 25635582 10.1097/MOP.0000000000000194 PMC4422489

[13] Yamamura T, Morisada N, Nozu K, Minamikawa S, Ishimori S, Toyoshima D, et al Rare renal ciliopathies in non-consanguineous families that were identified by targeted resequencing. Clinical and experimental nephrology. 2016 doi: 10.1007/s10157-016-1256-x . 26968886 10.1007/s10157-016-1256-x

[14] Schaefer E, Zaloszyc A, Lauer J, Durand M, Stutzmann F, Perdomo-Trujillo Y, et al Mutations in SDCCAG8/NPHP10 Cause Bardet-Biedl Syndrome and Are Associated with Penetrant Renal Disease and Absent Polydactyly. Molecular syndromology. 2011;1(6):273–81. Epub 2011/12/23. doi: 10.1159/000331268 . 22190896 10.1159/000331268 PMC3214956

[15] Kenedy AA, Cohen KJ, Loveys DA, Kato GJ, Dang CV. Identification and characterization of the novel centrosome-associated protein CCCAP. Gene. 2003;303:35–46. . 12559564 10.1016/s0378-1119(02)01141-1

[16] Airik R, Slaats GG, Guo Z, Weiss AC, Khan N, Ghosh A, et al Renal-retinal ciliopathy gene Sdccag8 regulates DNA damage response signaling. J Am Soc Nephrol. 2014;25(11):2573–83. doi: 10.1681/ASN.2013050565 . 24722439 10.1681/ASN.2013050565 PMC4214515

[17] Airik R, Schueler M, Airik M, Cho J, Ulanowicz KA, Porath JD, et al SDCCAG8 Interacts with RAB Effector Proteins RABEP2 and ERC1 and Is Required for Hedgehog Signaling. PLoS One. 2016;11(5):e0156081 doi: 10.1371/journal.pone.0156081 . 27224062 10.1371/journal.pone.0156081 PMC4880186

[18] Insolera R, Shao W, Airik R, Hildebrandt F, Shi SH. SDCCAG8 regulates pericentriolar material recruitment and neuronal migration in the developing cortex. Neuron. 2014;83(4):805–22. doi: 10.1016/j.neuron.2014.06.029 . 25088364 10.1016/j.neuron.2014.06.029 PMC4141904

[19] Coppieters F, Casteels I, Meire F, De Jaegere S, Hooghe S, van Regemorter N, et al Genetic screening of LCA in Belgium: predominance of CEP290 and identification of potential modifier alleles in AHI1 of CEP290-related phenotypes. Hum Mutat. 2010;31(10):E1709–66. doi: 10.1002/humu.21336 . 20683928 10.1002/humu.21336 PMC3048164

[20] Zaki MS, Sattar S, Massoudi RA, Gleeson JG. Co-occurrence of distinct ciliopathy diseases in single families suggests genetic modifiers. Am J Med Genet A. 2011;155A(12):3042–9. doi: 10.1002/ajmg.a.34173 . 22002901 10.1002/ajmg.a.34173 PMC3415794

[21] Abdelhamed ZA, Wheway G, Szymanska K, Natarajan S, Toomes C, Inglehearn C, et al Variable expressivity of ciliopathy neurological phenotypes that encompass Meckel-Gruber syndrome and Joubert syndrome is caused by complex de-regulated ciliogenesis, Shh and Wnt signalling defects. Hum Mol Genet. 2013;22(7):1358–72. doi: 10.1093/hmg/dds546 . 23283079 10.1093/hmg/dds546 PMC3596847

[22] Zhang Y, Seo S, Bhattarai S, Bugge K, Searby CC, Zhang Q, et al BBS mutations modify phenotypic expression of CEP290-related ciliopathies. Human molecular genetics. 2014;23(1):40–51. doi: 10.1093/hmg/ddt394 . 23943788 10.1093/hmg/ddt394 PMC3857943

[23] Masyukova SV, Landis DE, Henke SJ, Williams CL, Pieczynski JN, Roszczynialski KN, et al A Screen for Modifiers of Cilia Phenotypes Reveals Novel MKS Alleles and Uncovers a Specific Genetic Interaction between osm-3 and nphp-4. PLoS genetics. 2016;12(2):e1005841 doi: 10.1371/journal.pgen.1005841 . 26863025 10.1371/journal.pgen.1005841 PMC4749664

[24] Seo S, Baye LM, Schulz NP, Beck JS, Zhang Q, Slusarski DC, et al BBS6, BBS10, and BBS12 form a complex with CCT/TRiC family chaperonins and mediate BBSome assembly. Proceedings of the National Academy of Sciences of the United States of America. 2010;107(4):1488–93. doi: 10.1073/pnas.0910268107 . 20080638 10.1073/pnas.0910268107 PMC2824390

[25] Seo S, Zhang Q, Bugge K, Breslow DK, Searby CC, Nachury MV, et al A novel protein LZTFL1 regulates ciliary trafficking of the BBSome and Smoothened. PLoS genetics. 2011;7(11):e1002358 doi: 10.1371/journal.pgen.1002358 . 22072986 10.1371/journal.pgen.1002358 PMC3207910

[26] Livak KJ, Schmittgen TD. Analysis of relative gene expression data using real-time quantitative PCR and the 2(-Delta Delta C(T)) Method. Methods. 2001;25(4):402–8. doi: 10.1006/meth.2001.1262 . 11846609 10.1006/meth.2001.1262

[27] Lu B, Geurts AM, Poirier C, Petit DC, Harrison W, Overbeek PA, et al Generation of rat mutants using a coat color-tagged Sleeping Beauty transposon system. Mamm Genome. 2007;18(5):338–46. doi: 10.1007/s00335-007-9025-5 . 17557177 10.1007/s00335-007-9025-5

[28] Yang ZZ, Tschopp O, Di-Poi N, Bruder E, Baudry A, Dummler B, et al Dosage-dependent effects of Akt1/protein kinase Balpha (PKBalpha) and Akt3/PKBgamma on thymus, skin, and cardiovascular and nervous system development in mice. Molecular and cellular biology. 2005;25(23):10407–18. doi: 10.1128/MCB.25.23.10407-10418.2005 . 16287854 10.1128/MCB.25.23.10407-10418.2005 PMC1291243

[29] Easton RM, Cho H, Roovers K, Shineman DW, Mizrahi M, Forman MS, et al Role for Akt3/protein kinase Bgamma in attainment of normal brain size. Molecular and cellular biology. 2005;25(5):1869–78. doi: 10.1128/MCB.25.5.1869-1878.2005 . 15713641 10.1128/MCB.25.5.1869-1878.2005 PMC549378

[30] Warburton D, El-Hashash A, Carraro G, Tiozzo C, Sala F, Rogers O, et al Lung organogenesis. Curr Top Dev Biol. 2010;90:73–158. doi: 10.1016/S0070-2153(10)90003-3 . 20691848 10.1016/S0070-2153(10)90003-3 PMC3340128

[31] Davis RE, Swiderski RE, Rahmouni K, Nishimura DY, Mullins RF, Agassandian K, et al A knockin mouse model of the Bardet-Biedl syndrome 1 M390R mutation has cilia defects, ventriculomegaly, retinopathy, and obesity. Proceedings of the National Academy of Sciences of the United States of America. 2007;104(49):19422–7. doi: 10.1073/pnas.0708571104 . 18032602 10.1073/pnas.0708571104 PMC2148305

[32] Nishimura DY, Fath M, Mullins RF, Searby C, Andrews M, Davis R, et al Bbs2-null mice have neurosensory deficits, a defect in social dominance, and retinopathy associated with mislocalization of rhodopsin. Proceedings of the National Academy of Sciences of the United States of America. 2004;101(47):16588–93. doi: 10.1073/pnas.0405496101 15539463 10.1073/pnas.0405496101 PMC534519

[33] Swiderski RE, Nishimura DY, Mullins RF, Olvera MA, Ross JL, Huang J, et al Gene expression analysis of photoreceptor cell loss in bbs4-knockout mice reveals an early stress gene response and photoreceptor cell damage. Invest Ophthalmol Vis Sci. 2007;48(7):3329–40. doi: 10.1167/iovs.06-1477 . 17591906 10.1167/iovs.06-1477

[34] Fath MA, Mullins RF, Searby C, Nishimura DY, Wei J, Rahmouni K, et al Mkks-null mice have a phenotype resembling Bardet-Biedl syndrome. Hum Mol Genet. 2005;14(9):1109–18. doi: 10.1093/hmg/ddi123 . 15772095 10.1093/hmg/ddi123

[35] Loktev AV, Jackson PK. Neuropeptide Y family receptors traffic via the Bardet-Biedl syndrome pathway to signal in neuronal primary cilia. Cell Rep. 2013;5(5):1316–29. doi: 10.1016/j.celrep.2013.11.011 . 24316073 10.1016/j.celrep.2013.11.011

[36] Illman SA, Keski-Oja J, Parks WC, Lohi J. The mouse matrix metalloproteinase, epilysin (MMP-28), is alternatively spliced and processed by a furin-like proprotein convertase. Biochem J. 2003;375(Pt 1):191–7. doi: 10.1042/BJ20030497 . 12803542 10.1042/BJ20030497 PMC1223653

[37] Manicone AM, Harju-Baker S, Johnston LK, Chen AJ, Parks WC. Epilysin (matrix metalloproteinase-28) contributes to airway epithelial cell survival. Respir Res. 2011;12:144 doi: 10.1186/1465-9921-12-144 . 22040290 10.1186/1465-9921-12-144 PMC3225336

[38] Ma Y, Halade GV, Zhang J, Ramirez TA, Levin D, Voorhees A, et al Matrix metalloproteinase-28 deletion exacerbates cardiac dysfunction and rupture after myocardial infarction in mice by inhibiting M2 macrophage activation. Circ Res. 2013;112(4):675–88. doi: 10.1161/CIRCRESAHA.111.300502 . 23261783 10.1161/CIRCRESAHA.111.300502 PMC3597388

[39] Gharib SA, Johnston LK, Huizar I, Birkland TP, Hanson J, Wang Y, et al MMP28 promotes macrophage polarization toward M2 cells and augments pulmonary fibrosis. J Leukoc Biol. 2014;95(1):9–18. doi: 10.1189/jlb.1112587 . 23964118 10.1189/jlb.1112587 PMC3868192

[40] Manicone AM, Birkland TP, Lin M, Betsuyaku T, van Rooijen N, Lohi J, et al Epilysin (MMP-28) restrains early macrophage recruitment in Pseudomonas aeruginosa pneumonia. J Immunol. 2009;182(6):3866–76. doi: 10.4049/jimmunol.0713949 . 19265166 10.4049/jimmunol.0713949 PMC2721855

[41] Mavrommatis E, Fish EN, Platanias LC. The schlafen family of proteins and their regulation by interferons. J Interferon Cytokine Res. 2013;33(4):206–10. doi: 10.1089/jir.2012.0133 . 23570387 10.1089/jir.2012.0133 PMC3624771

[42] Bell TA, de la Casa-Esperon E, Doherty HE, Ideraabdullah F, Kim K, Wang Y, et al The paternal gene of the DDK syndrome maps to the Schlafen gene cluster on mouse chromosome 11. Genetics. 2006;172(1):411–23. doi: 10.1534/genetics.105.047118 . 16172501 10.1534/genetics.105.047118 PMC1456169

[43] Guilbault C, Martin P, Houle D, Boghdady ML, Guiot MC, Marion D, et al Cystic fibrosis lung disease following infection with Pseudomonas aeruginosa in Cftr knockout mice using novel non-invasive direct pulmonary infection technique. Lab Anim. 2005;39(3):336–52. doi: 10.1258/0023677054306944 . 16004694 10.1258/0023677054306944

[44] Sassano A, Mavrommatis E, Arslan AD, Kroczynska B, Beauchamp EM, Khuon S, et al Human Schlafen 5 (SLFN5) Is a Regulator of Motility and Invasiveness of Renal Cell Carcinoma Cells. Molecular and cellular biology. 2015;35(15):2684–98. doi: 10.1128/MCB.00019-15 . 26012550 10.1128/MCB.00019-15 PMC4524119

