# Supplementary figures and images for: Genotypic and phenotypic characterization of the Sdccag8Tn(sb-Tyr)2161B.CA1C2Ove mouse model
Source: PLoS One. 2018 Feb 14;13(2):e0192755. doi: 10.1371/journal.pone.0192755 (PMC5812623; doi:10.1371/journal.pone.0192755)

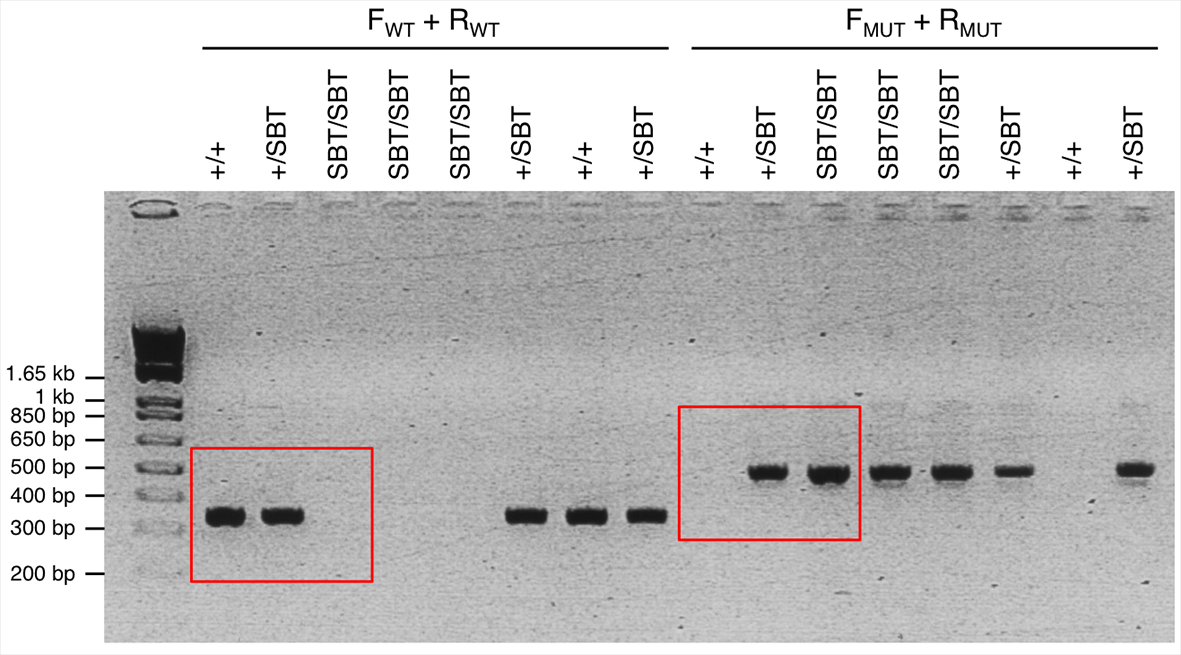

Supplement: S1 Fig — Red boxes denote DNA samples shown in Fig 2A. (TIF) [file pone.0192755.s001.tif]

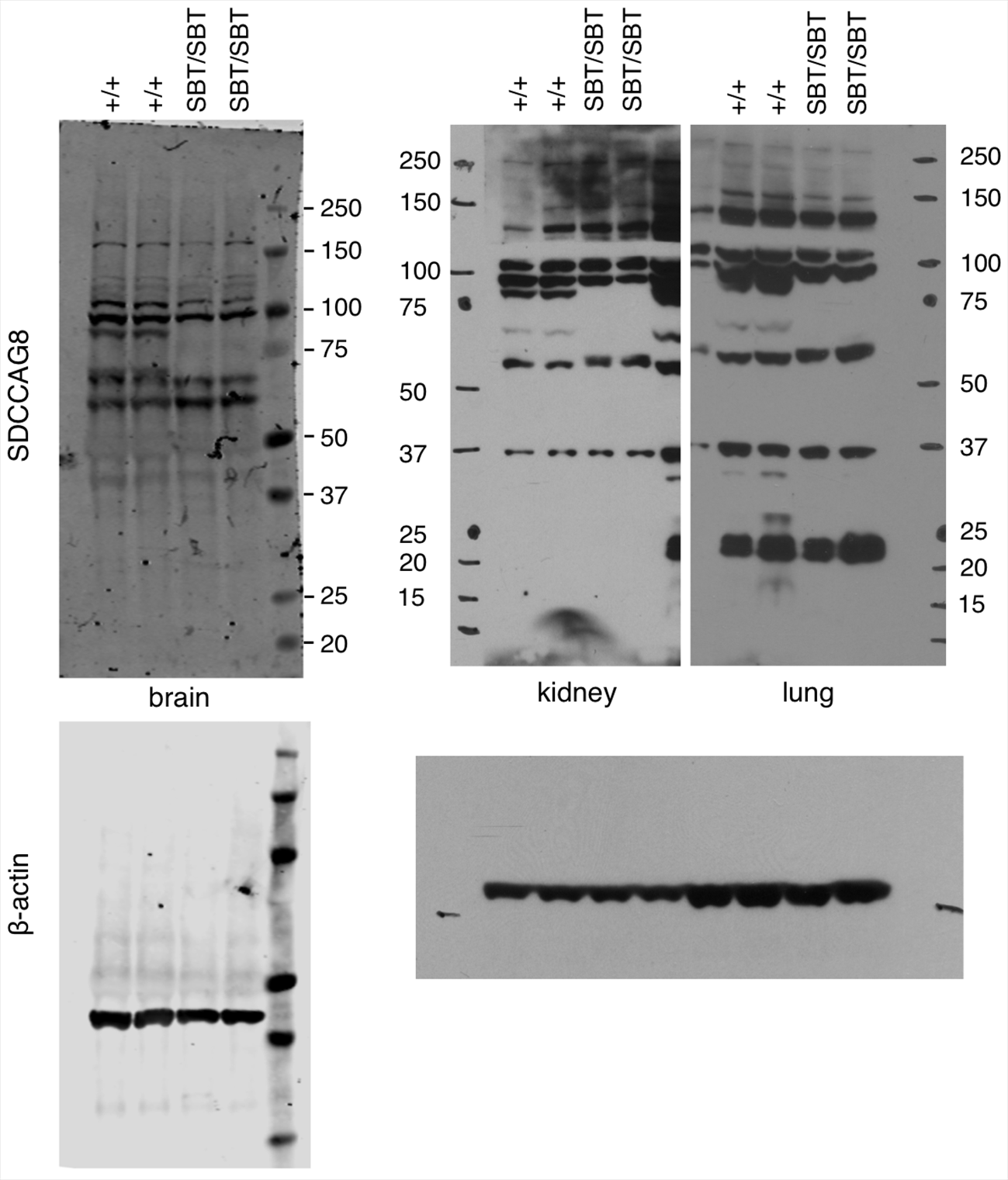

Supplement: S2 Fig — (TIF) [file pone.0192755.s002.tif]

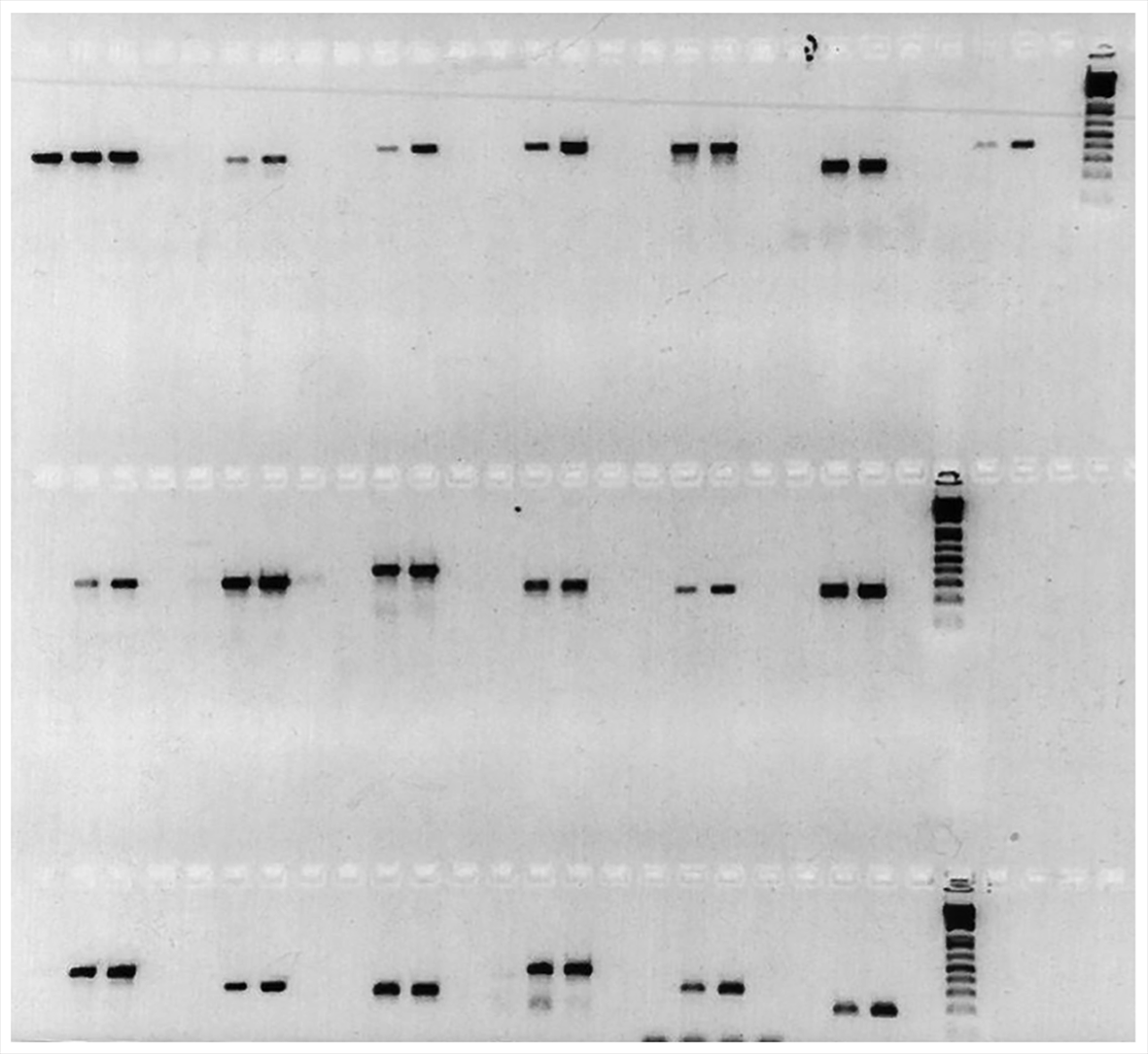

Supplement: S3 Fig — (TIF) [file pone.0192755.s003.tif]
