# Supplementary material for: Genotypic and phenotypic characterization of the Sdccag8Tn(sb-Tyr)2161B.CA1C2Ove mouse model
Source: PLoS One. 2018 Feb 14;13(2):e0192755. doi: 10.1371/journal.pone.0192755 (PMC5812623; doi:10.1371/journal.pone.0192755)
Supplement: S1 Table — (DOCX) [file pone.0192755.s004.docx]

**S1 Table S1: Genotyping PCR primers for *Sdccag8^SBT^***

| Gene | Primer Name | Direction | Sequence |
| --- | --- | --- | --- |
| *Sdccag8* | Fw | Forward | TGCTGTTTCCTCGTCACGTA |
| *Sdccag8* | Rw | Reverse | GCACCGTAAGACTGGCTCTC |
| *Sdccag8* | Fm | Forward | TCTAAGAAATGACTGGGATTTGC |
| *Sdccag8* | Rm | Reverse | CACCAAGTACCAGACGCTGA |
