# Supplementary material for: Genotypic and phenotypic characterization of the Sdccag8Tn(sb-Tyr)2161B.CA1C2Ove mouse model
Source: PLoS One. 2018 Feb 14;13(2):e0192755. doi: 10.1371/journal.pone.0192755 (PMC5812623; doi:10.1371/journal.pone.0192755)
Supplement: S2 Table — (DOCX) [file pone.0192755.s005.docx]

**S2 Table. Primers designed to amplify the exons of interest in *Sdccag8* and *Akt3.***

| Gene | Exon | Direction | Sequence |
| --- | --- | --- | --- |
| *Akt3* | 1 | Forward | CAAAGACTTAGTTGTTTGTTATTGCT |
| *Akt3* | 1 | Reverse | ATGGTAAAGTTTCTTTCAGCCCTA |
| *Akt3* | 2 | Forward | TTTTGCAGGTCAGTGTTAATGG |
| *Akt3* | 2 | Reverse | TGATCATTTCTTTCCATCAAGTG |
| *Akt3* | 3 | Forward | GACTGAAGATGGGCTCCAAA |
| *Akt3* | 3 | Reverse | CATGCAAGACAACAGAAACAGA |
| *Akt3* | 4 | Forward | AGGGATGTTGGAAGAGACTGTG |
| *Akt3* | 4 | Reverse | GTCCCACAAACATCCTGGAC |
| *Akt3* | 5 | Forward | TTGGAAATTGGTCTAGAAATTGG |
| *Akt3* | 5 | Reverse | CAAGAGAATAACAGTTTCACCATAAT |
| *Akt3* | 6 | Forward | AAGCAACATAGGAGGTGTACCA |
| *Akt3* | 6 | Reverse | TCATTCTATGCTAAAACCCCACT |
| *Akt3* | 7 | Forward | TCAGTCTCAGGTGACGCAGT |
| *Akt3* | 7 | Reverse | CTATGCAGACCTGCTGTGGA |
| *Akt3* | 8 | Forward | CACAATATGGTGATGAGAGGTG |
| *Akt3* | 8 | Reverse | TTCAGACCAACTACAGCAAACAA |
| *Akt3* | 9 | Forward | CCACGTCTCTTTCCAGTACCC |
| *Akt3* | 9 | Reverse | CCCATCATCTAAGTTAAAAGCAA |
| *Akt3* | 10 | Forward | TGATGACGATGACGACAAGG |
| *Akt3* | 10 | Reverse | TGCTTCCAGAAATCAGGCATA |
| *Akt3* | 11 | Forward | CATGGCTATGCATTTCAAAAA |
| *Akt3* | 11 | Reverse | TCAATTCCAAAGCAAAACAGAA |
| *Akt3* | 12 | Forward | CCTTACACAGGACGCACCTT |
| *Akt3* | 12 | Reverse | TTCAGAGTGCCATCAACTGC |
| *Akt3* | 13 | Forward | CTGTTTCTGAGTGTAGGCAGAGT |
| *Akt3* | 13 | Reverse | GGTGCCCCTGCTAACTGTAA |
| *Sdccag8* | 13 | Forward | CAATCAGATTTCTCACGATCCA |
| *Sdccag8* | 13 | Reverse | GCCTGAAAGCTGTGTGTGAA |
| *Sdccag8* | 14 | Forward | CTGCAGTCTGTGGTCTGTGG |
| *Sdccag8* | 14 | Reverse | GCCAAAGGTTACAGGTGTTCC |
| *Sdccag8* | 15 | Forward | GGAAGGGCAGAGTGATCTGA |
| *Sdccag8* | 15 | Reverse | AAAATGCAATTTTAAAGCCAAA |
| *Sdccag8* | 16 | Forward | ACCTCTCAGACCTCCCCAGT |
| *Sdccag8* | 16 | Reverse | CAATGCCTACCGTGTCATGT |
| *Sdccag8* | 17 | Forward | GCATTGGAACGTCAACACATA |
| *Sdccag8* | 17 | Reverse | CTGTGTGTGTTCCCACCCTA |
| *Sdccag8* | 18 | Forward | AGCTGACCAAACCAGCAAAT |
| *Sdccag8* | 18 | Reverse | GTGCACTGTGGCACATTAAG |
