# Supplementary material for: Genotypic and phenotypic characterization of the Sdccag8Tn(sb-Tyr)2161B.CA1C2Ove mouse model
Source: PLoS One. 2018 Feb 14;13(2):e0192755. doi: 10.1371/journal.pone.0192755 (PMC5812623; doi:10.1371/journal.pone.0192755)
Supplement: S3 Table — (DOCX) [file pone.0192755.s006.docx]

**S3 Table.** **Quantitative real time PCR primers used in this study**

| Gene | Primer location | Exon location | Direction | Sequence |
| --- | --- | --- | --- | --- |
| *Sdccag8* | c.1171-1271 | 10-11 | Forward | GCTGTGGAGAAGGAGATGATAAA |
| *Sdccag8* | c.1171-1271 | 10-11 | Reverse | GCCTCCAGCTTGGCAATA |
| *Sdccag8* | c.1705-1790 | 14-15 | Forward | CAGGAGCTGACACAGAAGATAC |
| *Sdccag8* | c.1705-1790 | 14-15 | Reverse | TTCTGGGATGTCAGCAGTAAAT |
| *Akt3* | c.237-259 | 3-4 | Forward | CAGAACGACCAAAGCCAAATAC |
| *Akt3* | c. 326-348 | 3-4 | Reverse | CTTCCGTCCACTCTTCTCTTTC |
