# Supplementary material for: Genotypic and phenotypic characterization of the Sdccag8Tn(sb-Tyr)2161B.CA1C2Ove mouse model
Source: PLoS One. 2018 Feb 14;13(2):e0192755. doi: 10.1371/journal.pone.0192755 (PMC5812623; doi:10.1371/journal.pone.0192755)
Supplement: S4 Table — (DOCX) [file pone.0192755.s007.docx]

**S4 Table. Primers designed to amplify SNPs of interest**

| Gene | Direction | Sequence |
| --- | --- | --- |
| rs26943877 | Forward | AGGCATGGGGAATGTGATAG |
| rs26943877 | Reverse | GCTGCAGAATGGGATCAGTA |
| rs3023266 | Forward | CCGAGCATGACTTTGACAGA |
| rs3023266 | Reverse | GCAACCCTACCCCAAGAAGT |
| rs26956597 | Forward | AACTGCACTTTGAGGCAGAAG |
| rs26956597 | Reverse | GGCAACAAGATGTCACCTCA |
| rs26915029 | Forward | CAGCGACAGAGTTCTTGCAT |
| rs26915029 | Reverse | CAGCCAAGGTAGCACACACA |
| rs3714172 | Forward | CAGGCTGGCCTTATAGCTTG |
| rs3714172 | Reverse | TCTTAGGCATGGAGGAATGC |
| rs28214055 | Forward | ATGGGCTGAGAGCAGATGAT |
| rs28214055 | Reverse | AGAGCCTGAACTGTGGGCTA |
| rs29432702 | Forward | GGAACAGGACTGCTTTCTGC |
| rs29432702 | Reverse | CGGACACTTCTCGTCTCCTC |
| rs6169904 | Forward | CTCCCCATTCCATCTCTGAA |
| rs6169904 | Reverse | CAAATCATCAGGAGGCCAGT |
| rs3141832 | Forward | ATACAGCACCCCAGCGTTAC |
| rs3141832 | Reverse | GGCAGAGGCAGTCAGATTTC |
| rs27100337 | Forward | AGGGAAATGAAGCAGGAGGT |
| rs27100337 | Reverse | ACAAGCCAAGAATCCACCAC |
| rs13481176 | Forward | TTGCTCTTCAGGTGGTCCAT |
| rs13481176 | Reverse | GGGAAGGGACTGAGTGTTGA |
