# Supplementary material for: Genotypic and phenotypic characterization of the Sdccag8Tn(sb-Tyr)2161B.CA1C2Ove mouse model
Source: PLoS One. 2018 Feb 14;13(2):e0192755. doi: 10.1371/journal.pone.0192755 (PMC5812623; doi:10.1371/journal.pone.0192755)
Supplement: S5 Table — Comparison of allele distribution in mice that died at P0 to mice that survived to P21 or greater as analyzed by a Chi Square test of independence. (DOCX) [file pone.0192755.s008.docx]

**S5 Table.** **A custom 150-SNP panel from Jackson Lab showed an association to survival at rs3023266 and rs3714172 (bold/italics).** Comparison of allele distribution in mice that died at P0 to mice that survived to P21 or greater as analyzed by a Chi Square test of independence

|  |  | Fatalities | | | Survivors | | |  |
| --- | --- | --- | --- | --- | --- | --- | --- | --- |
| SNP | Genomic location | FVB | 129 | Het | FVB | 129 | Het | p-value |
| rs3667401 | 01:007166135 | 10 | 0 | 0 | 10 | 0 | 0 | NA |
| rs4137502 | 01:031264126 | 3 | 2 | 5 | 5 | 2 | 3 | 0.840871591 |
| rs3654040 | 01:046003967 | 3 | 0 | 7 | 4 | 2 | 4 | 0.324486755 |
| rs3024048 | 01:067230857 | 3 | 1 | 6 | 5 | 3 | 2 | 0.297157028 |
| rs3677697 | 01:080181487 | 2 | 1 | 7 | 4 | 2 | 4 | 0.473205268 |
| rs3684654 | 01:106073928 | 4 | 1 | 5 | 5 | 2 | 3 | 0.712832872 |
| rs4222579 | 01:126655393 | 4 | 1 | 5 | 5 | 1 | 4 | 1 |
| rs3677464 | 01:139952425 | 6 | 0 | 4 | 6 | 0 | 4 | NA |
| rs3680033 | 01:150718101 | 7 | 0 | 3 | 6 | 0 | 4 | NA |
| rs13476265 | 01:180301121 | 10 | 0 | 0 | 10 | 0 | 0 | NA |
| rs4222922 | 01:194803746 | 6 | 0 | 4 | 10 | 0 | 0 | NA |
| rs3664986 | 02:005430515 | 6 | 2 | 2 | 2 | 1 | 7 | 0.117578824 |
| rs13476412 | 02:031721080 | 5 | 1 | 4 | 4 | 1 | 5 | 1 |
| rs13476474 | 02:047368792 | 5 | 1 | 4 | 4 | 1 | 5 | 1 |
| rs3022886 | 02:063356615 | 6 | 0 | 4 | 4 | 1 | 5 | 0.656683433 |
| rs4223212 | 02:072065558 | 5 | 0 | 5 | 4 | 1 | 5 | 1 |
| rs3682381 | 02:094964773 | 5 | 0 | 5 | 4 | 1 | 5 | 1 |
| rs4223406 | 02:114758266 | 5 | 0 | 5 | 4 | 1 | 5 | 1 |
| rs3662211 | 02:131136658 | 5 | 1 | 4 | 4 | 1 | 5 | 1 |
| rs3689600 | 02:159897340 | 5 | 1 | 4 | 4 | 1 | 5 | 1 |
| rs13476913 | 02:172504164 | 5 | 2 | 3 | 3 | 1 | 6 | 0.483945161 |
| rs4223706 | 03:007561998 | 4 | 1 | 5 | 2 | 2 | 6 | 0.701412986 |
| rs13477017 | 03:022347144 | 5 | 1 | 4 | 3 | 2 | 5 | 0.716102839 |
| rs3700620 | 03:035115028 | 4 | 2 | 4 | 3 | 2 | 5 | 1 |
| rs6246988 | 03:056067960 | 4 | 1 | 5 | 2 | 1 | 7 | 0.798722013 |
| rs3672565 | 03:081123258 | 10 | 0 | 0 | 10 | 0 | 0 | NA |
| rs6322792 | 03:095186319 | 10 | 0 | 0 | 10 | 0 | 0 | NA |
| rs4224164 | 03:115468851 | 4 | 1 | 5 | 6 | 3 | 1 | 0.206307937 |
| rs3677929 | 03:127463765 | 3 | 1 | 6 | 7 | 1 | 2 | 0.242607574 |
| rs3665359 | 03:152200034 | 4 | 3 | 3 | 6 | 1 | 3 | 0.632243678 |
| rs3694594 | 04:003163167 | 4 | 0 | 6 | 2 | 1 | 7 | 0.627843722 |
| rs3709725 | 04:017015246 | 6 | 0 | 4 | 2 | 1 | 7 | 0.169798302 |
| rs3703981 | 04:034965128 | 3 | 1 | 6 | 4 | 2 | 4 | 0.713412866 |
| rs4224501 | 04:055642665 | 4 | 1 | 5 | 5 | 1 | 4 | 1 |
| rs16265 | 04:075245266 | 3 | 2 | 5 | 5 | 1 | 4 | 0.714802852 |
| rs4139384 | 04:092052171 | 1 | 3 | 6 | 5 | 1 | 4 | 0.20801792 |
| rs4224736 | 04:117567852 | 2 | 4 | 4 | 5 | 1 | 4 | 0.287807122 |
| rs3711319 | 04:125988404 | 2 | 5 | 3 | 4 | 2 | 4 | 0.535174648 |
| rs13478034 | 04:143423535 | 2 | 4 | 4 | 4 | 2 | 4 | 0.483365166 |
| rs3693453 | 05:013136419 | 5 | 0 | 5 | 1 | 2 | 7 | 0.07698923 |
| rs4225179 | 05:035182717 | 4 | 0 | 6 | 2 | 3 | 5 | 0.31102689 |
| rs13478210 | 05:041667588 | 4 | 2 | 4 | 2 | 3 | 5 | 0.729052709 |
| rs13478287 | 05:061148036 | 10 | 0 | 0 | 10 | 0 | 0 | NA |
| rs4225300 | 05:076427870 | 3 | 1 | 6 | 3 | 3 | 4 | 0.632523675 |
| rs3712451 | 05:081041323 | 3 | 1 | 6 | 3 | 3 | 4 | 0.630903691 |
| rs3023049 | 05:104525294 | 4 | 2 | 4 | 2 | 1 | 7 | 0.475275247 |
| rs4225482 | 05:120623301 | 4 | 2 | 4 | 2 | 1 | 7 | 0.47395526 |
| rs3713156 | 05:145143884 | 1 | 5 | 4 | 3 | 2 | 5 | 0.281087189 |
| rs3701161 | 06:010133483 | 1 | 4 | 5 | 3 | 0 | 7 | 0.162288377 |
| rs3684277 | 06:023207025 | 1 | 4 | 5 | 3 | 1 | 6 | 0.249227508 |
| rs6266537 | 06:038230055 | 1 | 2 | 7 | 4 | 1 | 5 | 0.443845562 |
| rs4225867 | 06:048811360 | 1 | 2 | 7 | 4 | 1 | 5 | 0.440155598 |
| rs3721547 | 06:078048356 | 1 | 3 | 6 | 4 | 1 | 5 | 0.251147489 |
| rs3683775 | 06:091998787 | 3 | 2 | 5 | 4 | 1 | 5 | 1 |
| rs3665833 | 06:106057600 | 3 | 3 | 4 | 3 | 1 | 6 | 0.629153708 |
| rs3684245 | 06:126258422 | 2 | 3 | 5 | 4 | 3 | 3 | 0.848361516 |
| rs3688082 | 06:146079591 | 3 | 4 | 3 | 2 | 4 | 4 | 1 |
| rs3670158 | 07:006208019 | 1 | 5 | 4 | 1 | 4 | 5 | 1 |
| rs3675839 | 07:011167675 | 1 | 6 | 3 | 1 | 4 | 5 | 0.809381906 |
| rs3675009 | 07:029991530 | 0 | 4 | 6 | 0 | 4 | 6 | NA |
| rs13479279 | 07:047745716 | 1 | 3 | 6 | 2 | 2 | 6 | 1 |
| rs3679035 | 07:065183517 | 3 | 3 | 4 | 2 | 2 | 6 | 0.727832722 |
| rs3654319 | 07:083056176 | 3 | 4 | 3 | 2 | 1 | 7 | 0.272127279 |
| rs3681072 | 07:102009856 | 3 | 4 | 3 | 3 | 0 | 7 | 0.074629254 |
| rs13479502 | 07:112553982 | 3 | 3 | 4 | 3 | 1 | 6 | 0.630783692 |
| rs13479544 | 07:124000818 | 2 | 4 | 4 | 4 | 2 | 4 | 0.484125159 |
| rs3023878 | 08:003369779 | 4 | 2 | 4 | 3 | 1 | 6 | 0.712722873 |
| rs3653751 | 08:032986730 | 5 | 2 | 3 | 2 | 2 | 6 | 0.396886031 |
| rs3661085 | 08:054093936 | 3 | 2 | 5 | 3 | 4 | 3 | 0.848631514 |
| rs4227196 | 08:065849115 | 4 | 1 | 5 | 3 | 4 | 3 | 0.395666043 |
| rs3023196 | 08:088063837 | 3 | 1 | 6 | 3 | 3 | 4 | 0.631603684 |
| rs3726020 | 08:101099186 | 2 | 1 | 7 | 4 | 3 | 3 | 0.285527145 |
| rs3686697 | 08:117163724 | 1 | 3 | 6 | 3 | 0 | 7 | 0.190678093 |
| rs3719348 | 09:016198772 | 1 | 3 | 6 | 4 | 0 | 6 | 0.094669053 |
| rs3023203 | 09:029233439 | 1 | 3 | 6 | 3 | 1 | 6 | 0.409135909 |
| rs3690374 | 09:039909037 | 1 | 5 | 4 | 4 | 1 | 5 | 0.136148639 |
| rs3656848 | 09:063923771 | 2 | 4 | 4 | 5 | 1 | 4 | 0.288887111 |
| rs3673117 | 09:086008489 | 1 | 2 | 7 | 5 | 2 | 3 | 0.151758482 |
| rs3685576 | 09:110057634 | 2 | 2 | 6 | 5 | 2 | 3 | 0.394556054 |
| rs4135782 | 10:008017793 | 2 | 1 | 7 | 3 | 3 | 4 | 0.50099499 |
| rs3023233 | 10:028554348 | 2 | 1 | 7 | 4 | 3 | 3 | 0.285687143 |
| rs3661754 | 10:045078228 | 2 | 2 | 6 | 2 | 3 | 5 | 1 |
| rs13480660 | 10:077371167 | 4 | 3 | 3 | 4 | 2 | 4 | 1 |
| rs4228444 | 10:099683776 | 4 | 3 | 3 | 3 | 3 | 4 | 1 |
| rs3674646 | 10:120289167 | 4 | 3 | 3 | 2 | 1 | 7 | 0.284147159 |
| rs4222040 | 11:005661856 | 3 | 0 | 7 | 3 | 3 | 4 | 0.239157608 |
| rs4228622 | 11:022864828 | 4 | 0 | 6 | 1 | 3 | 6 | 0.095739043 |
| rs3723833 | 11:033040833 | 4 | 0 | 6 | 1 | 3 | 6 | 0.093839062 |
| ***rs3023266*** | ***11:061500282*** | ***4*** | ***1*** | ***5*** | ***0*** | ***6*** | ***4*** | ***0.017669823*** |
| ***rs3714172*** | ***11:076223449*** | ***4*** | ***2*** | ***4*** | ***0*** | ***7*** | ***3*** | ***0.030469695*** |
| rs13481176 | 11:098391535 | 4 | 3 | 3 | 1 | 5 | 4 | 0.395146049 |
| rs3023710 | 11:118804416 | 2 | 3 | 5 | 1 | 4 | 5 | 1 |
| rs4140017 | 12:011234798 | 1 | 5 | 4 | 2 | 2 | 6 | 0.475705243 |
| rs3089465 | 12:031510473 | 1 | 4 | 5 | 2 | 2 | 6 | 0.699123009 |
| rs3688316 | 12:049281625 | 1 | 4 | 5 | 1 | 1 | 8 | 0.444555554 |
| rs3023345 | 12:064212930 | 10 | 0 | 0 | 10 | 0 | 0 | NA |
| rs3700554 | 12:071296001 | 0 | 0 | 10 | 0 | 0 | 10 | NA |
| rs13481630 | 12:099526845 | 0 | 6 | 4 | 0 | 4 | 6 | NA |
| rs3691931 | 12:106729531 | 1 | 3 | 6 | 3 | 3 | 4 | 0.63398366 |
| rs4229629 | 13:010476978 | 5 | 0 | 5 | 2 | 4 | 4 | 0.102418976 |
| rs3654710 | 13:030913320 | 3 | 0 | 7 | 2 | 4 | 4 | 0.150938491 |
| rs3676930 | 13:047833889 | 10 | 0 | 0 | 10 | 0 | 0 | NA |
| rs3023383 | 13:059590161 | 2 | 2 | 6 | 2 | 3 | 5 | 1 |
| rs3673712 | 13:077818021 | 0 | 3 | 7 | 3 | 3 | 4 | 0.239127609 |
| rs13481932 | 13:085133616 | 1 | 3 | 6 | 3 | 2 | 5 | 0.702382976 |
| rs4230010 | 13:093046894 | 1 | 2 | 7 | 3 | 1 | 6 | 0.664553354 |
| rs4230084 | 13:110566702 | 2 | 2 | 6 | 3 | 1 | 6 | 1 |
| rs4230113 | 13:112696462 | 2 | 3 | 5 | 2 | 1 | 7 | 0.819311807 |
| rs3669009 | 14:005898912 | 1 | 2 | 7 | 2 | 1 | 7 | 1 |
| rs13482109 | 14:022368155 | 1 | 2 | 7 | 1 | 1 | 8 | 1 |
| rs13482141 | 14:033881274 | 2 | 3 | 5 | 1 | 2 | 7 | 0.699913001 |
| rs3660830 | 14:055918382 | 1 | 2 | 7 | 1 | 2 | 7 | 1 |
| rs3676913 | 14:079218045 | 2 | 1 | 7 | 3 | 3 | 4 | 0.500744993 |
| rs3656066 | 14:096127583 | 3 | 1 | 6 | 3 | 3 | 4 | 0.631803682 |
| rs3707842 | 14:110887447 | 3 | 2 | 5 | 3 | 3 | 4 | 1 |
| rs3715343 | 15:005994001 | 3 | 3 | 4 | 2 | 2 | 6 | 0.726562734 |
| rs13482497 | 15:028279166 | 2 | 4 | 4 | 2 | 3 | 5 | 1 |
| rs3090057 | 15:041919640 | 3 | 1 | 6 | 3 | 3 | 4 | 0.634203658 |
| rs4222080 | 15:055206736 | 3 | 1 | 6 | 2 | 2 | 6 | 1 |
| rs13482635 | 15:069647296 | 2 | 1 | 7 | 2 | 2 | 6 | 1 |
| rs3690689 | 15:087100507 | 2 | 1 | 7 | 2 | 4 | 4 | 0.356226438 |
| rs3023429 | 15:102788257 | 2 | 3 | 5 | 2 | 4 | 4 | 1 |
| rs4153115 | 16:003310511 | 2 | 3 | 5 | 2 | 4 | 4 | 1 |
| rs4165029 | 16:015729768 | 10 | 0 | 0 | 10 | 0 | 0 | NA |
| rs4172707 | 16:035239412 | 3 | 4 | 3 | 1 | 2 | 7 | 0.286857131 |
| rs4186333 | 16:051080938 | 3 | 4 | 3 | 1 | 3 | 6 | 0.522524775 |
| rs4197785 | 16:068107787 | 3 | 2 | 5 | 1 | 3 | 6 | 0.697623024 |
| rs4219612 | 16:093066825 | 3 | 3 | 4 | 2 | 5 | 3 | 0.736132639 |
| rs3664721 | 17:013493244 | 3 | 2 | 5 | 2 | 3 | 5 | 1 |
| rs3696834 | 17:028172639 | 3 | 1 | 6 | 2 | 2 | 6 | 1 |
| rs6395893 | 17:037829090 | 3 | 1 | 6 | 1 | 2 | 7 | 0.666593334 |
| rs3710084 | 17:057262935 | 3 | 1 | 6 | 2 | 3 | 5 | 0.700452995 |
| rs3090282 | 17:065004394 | 0 | 6 | 4 | 0 | 9 | 1 | NA |
| rs3659009 | 17:089136255 | 3 | 4 | 3 | 2 | 3 | 5 | 0.733882661 |
| rs4231742 | 18:010953833 | 1 | 3 | 6 | 3 | 2 | 5 | 0.699133009 |
| rs3714233 | 18:029963387 | 0 | 4 | 6 | 4 | 2 | 4 | 0.107508925 |
| rs3695510 | 18:039129483 | 0 | 4 | 6 | 4 | 3 | 3 | 0.134108659 |
| rs3089229 | 18:055728214 | 1 | 5 | 4 | 4 | 2 | 4 | 0.288947111 |
| rs3668697 | 18:075088655 | 1 | 3 | 6 | 1 | 3 | 6 | 1 |
| rs3670234 | 18:086980249 | 2 | 3 | 5 | 1 | 2 | 7 | 0.699413006 |
| rs3023477 | 19:003607482 | 2 | 2 | 6 | 2 | 1 | 7 | 1 |
| rs3692733 | 19:013339654 | 3 | 3 | 4 | 2 | 1 | 7 | 0.50302497 |
| rs13483600 | 19:034003337 | 3 | 2 | 5 | 3 | 3 | 4 | 1 |
| rs4139797 | 19:044978078 | 2 | 2 | 6 | 2 | 4 | 4 | 0.836561634 |
| rs3023498 | 19:060823449 | 2 | 3 | 5 | 2 | 3 | 5 | 1 |
