# Supplementary material for: Genotypic and phenotypic characterization of the Sdccag8Tn(sb-Tyr)2161B.CA1C2Ove mouse model
Source: PLoS One. 2018 Feb 14;13(2):e0192755. doi: 10.1371/journal.pone.0192755 (PMC5812623; doi:10.1371/journal.pone.0192755)
Supplement: S6 Table — (DOCX) [file pone.0192755.s009.docx]

**S6 Table.** **List of genes with chromosome position in the 8 Mb region between SNPs rs3714172 and rs3141832**

| Gene name | Genomic Start Site | Strand |
| --- | --- | --- |
| *Pitpna* | 11:75588078 | + |
| *Inpp5k* | 11:75631019 | + |
| *Myo1c* | 11:75651508 | + |
| *Crk* | 11:75679258 | + |
| *Ywhae* | 11:75732886 | + |
| *Doc2b* | 11:75768270 | - |
| *Gm12339* | 11:75795808 | + |
| *Rph3al* | 11:75899724 | - |
| *1700016K19Rik* | 11:75999911 | + |
| *Rflnb* | 11:76019194 | - |
| *Vps53* | 11:76046225 | - |
| *Fam57a* | 11:76202055 | + |
| *Gemin4* | 11:76210570 | - |
| *Dbil5* | 11:76217612 | + |
| *Glod4* | 11:76220394 | - |
| *Mrm3* | 11:76243735 | + |
| *Nxn* | 11:76257225 | - |
| *Timm22* | 11:76406924 | + |
| *Abr* | 11:76416791 | - |
| *Bhlha9* | 11:76672469 | + |
| *Tusc5* | 11:76679872 | + |
| *Gosr1* | 11:76726601 | - |
| *Cpd* | 11:76777207 | - |
| *Tmigd1* | 11:76904544 | + |
| *Blmh* | 11:76945655 | + |
| *Slc6a4* | 11:76998596 | + |
| *Nsrp1* | 11:77044291 | - |
| *Mir423* | 11:77078063 | - |
| *Efcab5* | 11:77089914 | - |
| *Ssh2* | 11:77216424 | + |
| *Coro6* | 11:77463912 | + |
| *Ankrd13b* | 11:77470486 | - |
| *Git1* | 11:77493411 | + |
| *Trp53i13* | 11:77508098 | - |
| *Abhd15* | 11:77515116 | + |
| *Taok1* | 11:77529161 | - |
| *Nufip2* | 11:77686138 | + |
| *Cryba1* | 11:77718613 | - |
| *Myo18a* | 11:77801297 | + |
| *Pipox* | 11:77880614 | - |
| *Gm11190* | 11:77912126 | - |
| *Sez6* | 11:77930838 | + |
| *Phf12* | 11:77982815 | + |
| *Dhrs13* | 11:78032312 | + |
| *Flot2* | 11:78040303 | + |
| *Mir144* | 11:78073004 | + |
| *Mir451b* | 11:78073169 | - |
| *Mir451a* | 11:78073169 | + |
| *Eral1* | 11:78073375 | - |
| *Fam222b* | 11:78094672 | + |
| *Traf4* | 11:78158422 | - |
| *Nek8* | 11:78166105 | - |
| *Tlcd1* | 11:78178657 | + |
| *Mir7653* | 11:78178826 | + |
| *Rpl23a* | 11:78180935 | - |
| *Snord42a* | 11:78181301 | - |
| *Snord4a* | 11:78181686 | - |
| *Snord42b* | 11:78183058 | - |
| *Rab34* | 11:78188426 | + |
| *Proca1* | 11:78193391 | + |
| *Supt6* | 11:78206748 | - |
| *Sdf2* | 11:78245745 | + |
| *2610507B11Rik* | 11:78261753 | + |
| *BC030499* | 11:78290840 | + |
| *Spag5* | 11:78301590 | + |
| *Aldoc* | 11:78323072 | + |
| *Pigs* | 11:78328421 | + |
| *Unc119* | 11:78343494 | + |
| *Foxn1* | 11:78357576 | - |
| *Slc13a2os* | 11:78394484 | + |
| *Slc13a2* | 11:78397275 | - |
| *Slc46a1* | 11:78465700 | + |
| *Sarm1* | 11:78472329 | - |
| *Vtn* | 11:78499119 | + |
| *Sebox* | 11:78503512 | + |
| *Tmem199* | 11:78507054 | - |
| *Poldip2* | 11:78512295 | + |
| *Tnfaip1* | 11:78522849 | - |
| *Ift20* | 11:78536435 | + |
| *Tmem97* | 11:78541816 | - |
| *Nlk* | 11:78567167 | - |
| *Fam58b* | 11:78750505 | - |
| *Lyrm9* | 11:78826594 | + |
| *Nos2* | 11:78920786 | + |
| *Lgals9* | 11:78962978 | - |
| *Ksr1* | 11:79013439 | - |
| *Gm11201* | 11:79023596 | + |
| *Wsb1* | 11:79239382 | - |
| *Nf1* | 11:79339891 | + |
| *AU040972* | 11:79481722 | - |
| *Omg* | 11:79500981 | - |
| *Evi2* | 11:79513384 | - |
| *Evi2b* | 11:79513384 | - |
| *Evi2a* | 11:79526560 | - |
| *Rab11fip4* | 11:79591211 | + |
| *Rab11fip4os1* | 11:79607078 | - |
| *Rab11fip4os2* | 11:79670372 | - |
| *Mir193a* | 11:79711968 | + |
| *Mir365-2* | 11:79726399 | + |
| *Utp6* | 11:79933955 | - |
| *Suz12* | 11:79993105 | + |
| *Crlf3* | 11:80046492 | - |
| *Atad5* | 11:80089399 | + |
| *Tefm* | 11:80136677 | - |
| *Adap2* | 11:80154161 | + |
| *Rnf135* | 11:80183871 | + |
| *Rhot1* | 11:80209054 | + |
| *Rhbdl3* | 11:80300911 | + |
| *5730455P16Rik* | 11:80360491 | - |
| *Zfp207* | 11:80383278 | + |
| *Psmd11* | 11:80428614 | + |
| *Cdk5r1* | 11:80477022 | + |
| *Myo1d* | 11:80482126 | - |
| *C030013C21Rik* | 11:80507409 | - |
| *Tmem98* | 11:80810414 | + |
| *4930507D10Rik* | 11:80850380 | - |
| *Spaca3* | 11:80858388 | + |
| *Asic2* | 11:80880162 | - |
| *1700071K01Rik* | 11:81572501 | - |
| *5530401A14Rik* | 11:81860676 | + |
| *Ccl2* | 11:82035576 | + |
| *Ccl7* | 11:82045711 | + |
| *Ccl11* | 11:82057831 | + |
| *Ccl12* | 11:82101844 | + |
| *Ccl8* | 11:82115184 | + |
| *Ccl1* | 11:82176665 | - |
| *Tmem132e* | 11:82388899 | + |
| *Gm11426* | 11:82633352 | + |
| *Cct6b* | 11:82719247 | - |
| *Zfp830* | 11:82764344 | + |
| *Gm11423* | 11:82779600 | - |
| *Lig3* | 11:82781108 | + |
| *Rffl* | 11:82803818 | - |
| *Rad51d* | 11:82871960 | - |
| *Fndc8* | 11:82892144 | + |
| *Nle1* | 11:82900767 | - |
| *Unc45b* | 11:82911252 | + |
| *Slfn5os* | 11:82942340 | - |
| *Slfn5* | 11:82952101 | + |
| *Slfn9* | 11:82980302 | - |
| *Mir7679* | 11:82985002 | - |
| *Slfn8* | 11:83002157 | - |
| *Slfn10-ps* | 11:83028125 | - |
| *Slfn2* | 11:83065111 | + |
| *Slfn1* | 11:83116844 | + |
| *Slfn4* | 11:83175185 | + |
| *Slfn3* | 11:83191329 | + |
| *AI662270* | 11:83223575 | + |
| *Slfn14* | 11:83275111 | - |
| *AA465934* | 11:83291698 | + |
| *AI450353* | 11:83293429 | + |
| *Snord7* | 11:83294303 | + |
| *Pex12* | 11:83294644 | - |
| *Ap2b1* | 11:83302696 | + |
| *Rasl10b* | 11:83410071 | + |
| *Gas2l2* | 11:83421634 | - |
| *1700020L24Rik* | 11:83437693 | + |
| *Mmp28* | 11:83440767 | - |
| *Taf15* | 11:83473107 | + |
| *Heatr9* | 11:83511678 | - |
| *Ccl5* | 11:83525778 | - |
| *Ccl9* | 11:83572916 | - |
